# Ecological and evolutionary dynamics of cell-virus-virophage systems

**DOI:** 10.1371/journal.pcbi.1010925

**Published:** 2024-02-20

**Authors:** Jose Gabriel Nino Barreat, Aris Katzourakis

**Affiliations:** Department of Biology, University of Oxford, Oxford, United Kingdom; Washington State University, UNITED STATES

## Abstract

Microbial eukaryotes, giant viruses and virophages form a unique hyperparasitic system. Virophages are parasites of the virus transcription machinery and can interfere with virus replication, resulting in a benefit to the eukaryotic host population. Surprisingly, virophages can integrate into the genomes of their cell or virus hosts, and have been shown to reactivate during coinfection. This raises questions about the role of integration in the dynamics of cell-virus-virophage systems. We use mathematical models and computational simulations to understand the effect of virophage integration on populations of cells and viruses. We also investigate multicellularity and programmed cell-death (PCD) as potential antiviral defence strategies used by cells. We found that virophages which enter the cell independently of the host virus, such as Mavirus, are expected to integrate commonly into the genomes of their cell hosts. Our models suggest that integrations from virophages without an independent mode of entry like Sputnik, are less likely to become fixed in the cell host population. Alternatively, we found that Sputnik virophages can stably persist integrated in the virus population, as long as they do not completely inhibit virus replication. We also show that increasing virophage inhibition can stabilise oscillatory dynamics, which may explain the long-term persistence of viruses and virophages in the environment. Our results demonstrate that inhibition by virophages and multicellularity are effective antiviral strategies that may act in synergy against viral infection in microbial species.

## Author summary

Giant viruses can be infected by their own viruses called virophages (‘virus eater’). Together with microbial eukaryotes, giant viruses and virophages form a unique parasitic system. Recently, virophages were discovered to become part of the genome of their host eukaryote or giant virus (a process called integration). We used mathematical models and simulations to explore how populations of cells, viruses and virophages interact in the presence of integration. We found that inhibition of virus replication by virophages can lead to stable interactions, and that cells and viruses carrying a virophage can become firmly established in the population, even when there is no direct advantage to the host. Computational simulations also indicated that inhibition by virophages and multicellularity can be protective for the cell population. Given that giant viruses and virophages seem to be at least as old as the early eukaryotes, our results could provide insights into the antiviral strategies used by ancient eukaryotic organisms.

## Introduction

Giant viruses are large dsDNA viruses from multiple families that belong to the clade of Nucleocytoplasmic Large DNA Viruses (NCLDVs) [1]. They are known to infect a wide variety of eukaryotes including algae, protozoans and vertebrates [1], and some are amongst the largest viruses known in terms of genome size and physical dimensions; which can be easily seen under the light microscope [2–4]. The striking sizes of giant viruses, which rival those of bacteria, are believed to have evolved independently from smaller viral ancestors via extensive horizontal gene transfer [5,6]. A distinctive feature in the infectious cycle of giant viruses is the formation of electron-dense areas in the cell cytoplasm where viral transcription and assembly take place [7]. These viral factories concentrate significant cell resources and are the sites where virally-encoded components of the transcription and translation machineries are expressed [8]. The newly assembled viruses exit by lysis or budding, which results in the death of the infected cell [9].

Virophages are viral parasites of giant viruses which use their viral factories for replication. The first virophage to be described was Sputnik, found in association with *Acanthamoeba polyphaga mimivirus* [10]. Sputnik could not replicate in the absence of its host virus and when grown together, caused the appearance of abortive and misassembled virus particles [10]. Another class of virophages was then discovered in the flagellate *Cafeteria burkhardae* (formerly *Cafeteria roenbergensis*) infected by *Cafeteria roenbergensis virus* (CroV) [11]. Similar to Sputnik, Mavirus required coinfection of the cell with CroV and interfered with CroV replication [11]. The Guarani virophage was also shown to have this inhibitory effect on the replication of its mimivirus host [12]. In general, it seems that virophages benefit their eukaryotic hosts by lowering the giant virus progeny and increasing the survival of the cell population [12,13]. However, this might not be universal as the Zamilon virophage does not seem to have a significant impact on its host virus [14], and so it is a “neutral” virophage.

Virophages can have different biological properties which are illustrated by Mavirus and Sputnik. Mavirus can infect the cell independently of its host virus via receptor-mediated endocytosis [11], while Sputnik enters the cell forming a complex with its host mimivirus by attaching to glycosylated protein fibres of the outer capsid [15,16]. Virophages can also integrate using different enzymes: a tyrosine recombinase in Sputnik or a rve-integrase in Mavirus [17]. Interestingly, only Mavirus-like virophages have been found in the genomes of their eukaryotic hosts [13,18]. Sputnik-like virophages have been found integrated in the genomes of the giant virus but not in the eukaryotic host, despite attempts to find integrated virophages in several *Acanthamoeba* species [19,20]. In the flagellate *Cafeteria*, reactivation of integrated Mavirus virophages works as an inducible defence system against CroV infection [13]. Although cells with integrated virophages lyse as a result of infection by the giant virus, exogenous virophages are produced which can interfere with viral replication in new rounds of coinfection, protecting the wider host population [13].

Giant viruses and virophages are emerging as important players in the function of aquatic ecosystems, as evidenced by their sheer abundance and diversity across globally distributed metagenomes [21–24]. They also seem to be ancient components of the eukaryotic virome [25–27], and could thus be implicated in the evolution of antiviral strategies used by the early eukaryotes. Here we use mathematical models and computational simulations to study the ecological and evolutionary dynamics of this tripartite parasitic system. Importantly, we consider the role of virophage integration on the system dynamics which had not been studied in previous theoretical work. Our models explain why different virophages follow different integration strategies, they show that virus inhibition by virophages can stabilise the dynamics of the system and that inhibitory virophages, multicellularity and programmed-cell death may be used as effective antiviral strategies. Taken together, our findings shed light on various aspects of the ecology and evolutionary genomics of virophages and their hosts, and provide a framework of testable hypotheses to inspire future experimental work.

## Results

### ODE models

We developed two ODE models of Mavirus and Sputnik integration into cells (See Materials and methods). First, we examined the system dynamics in the absence of virophages (S1 Fig). In both models, cells and viruses engaged in oscillatory predator-prey-like interactions. This oscillatory behaviour has also been observed in other models where cells are killed by lytic viruses [24,28,29]. In contrast to Lotka-Volterra predator-prey models where trajectories converge to different orbits depending on initial conditions (neutral oscillations), our models show trajectories that converge to a limit cycle attractor in 2 dimensions (a stable oscillatory regime). This is similar to the behaviour observed in Holling-Tanner predator-prey models where population growth is also controlled by a logistic term [30,31]. The appearance of an attractor is a desirable property since the system outcome is robust to small perturbations in the initial conditions [30].

**Fig 1:**
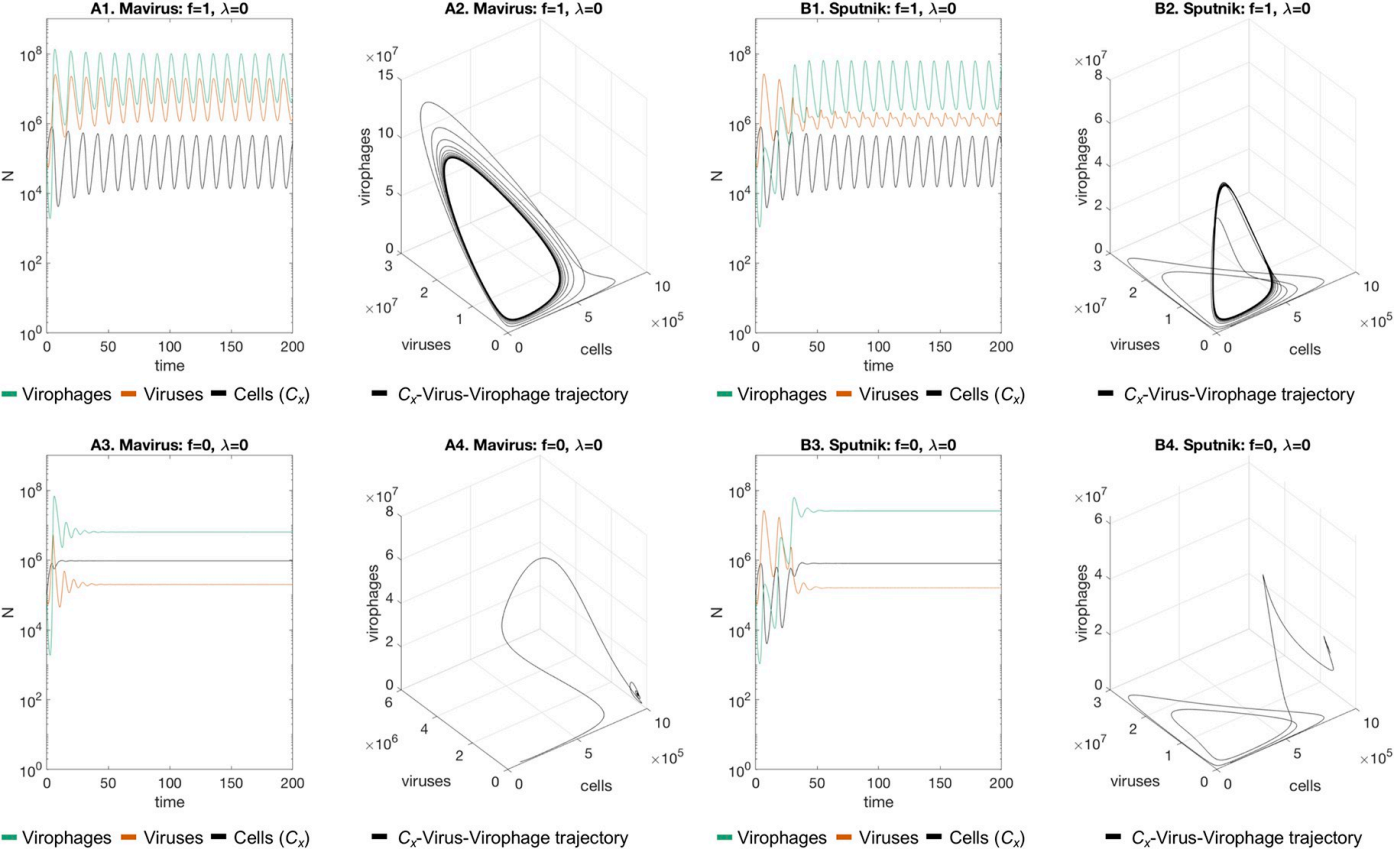
Dynamics of the Mavirus and Sputnik 1a models in the absence of virophage integration (baseline scenario). **A1-A2. Mavirus model:** interactions with neutral virophages (f = 1) produce oscillations which converge to a 3-dimensional limit cycle in state space**. B1-B2. Sputnik model:** a similar pattern is observed when virophages are neutral. **A3-A4, B3-B4:** introduction of totally inhibitory virophages (f = 0), stabilises dynamics to a point equilibrium in both models. Parameters for model 1: **α**_**1**_ = 1, **α**_**2**_ = 0.9, **β**_**1**_ = 10^−7^, **β**_**2**_ = 10^−6^, **γ**_**1**_ = 1.2, **γ**_**2**_ = 2.6, **λ** = 0, **φ**_**1**_ = 100, **φ**_**2**_ = 1000, **f** = {1, 0}, **K** = 10^6^, **r**_**1**_ = 1, **r**_**2**_ = 0.8, **p** = 0. Parameters for model 2 are the same except for **β**_**2**_ = 10^−7^ and **k** = 8⋅10^−7^. Initial conditions: **C**_**x,0**_ = 10^5^, **G**_**0**_ = 10^5^, **V** = 10^5^.

When we added neutral virophages to the system (which do not inhibit virus replication), all three populations showed a stable oscillatory regime (Fig 1A1-A2 and Fig 1B1-B2). This can be seen in the appearance in state-space of a 3-dimensional limit cycle attractor. Interestingly, when we set the inhibition parameter equal to zero (**f** = 0, virophages completely inhibit virus replication), the trajectories converge to a point equilibrium where the populations of cells, viruses and virophages remain unchanged (Fig 1A3-A4 and Fig 1B3-B4). This is an example of a Hopf bifurcation, where a fixed-point equilibrium gives rise to a limit cycle attractor [30,31]; in this case, as a function of the inhibition parameter (**f**).

### The effect of virophage integration into the cell genome

**Fig 2:**
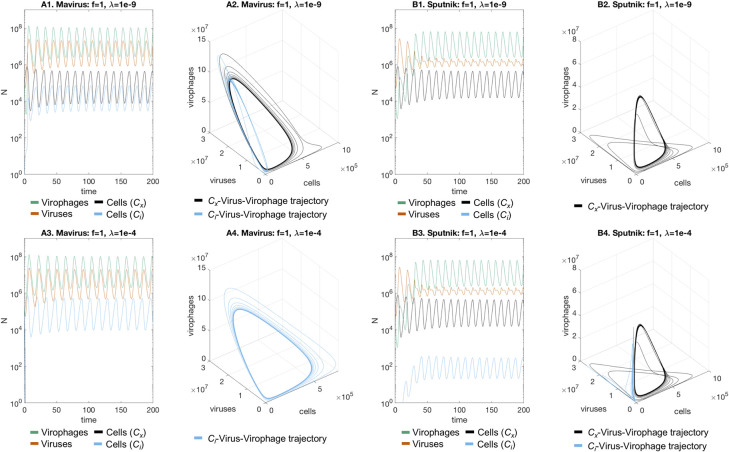
Dynamics of the Mavirus and Sputnik 1a models in the presence of virophage integration (neutral virophages). **A1-A2. Mavirus model:** in the presence of neutral virophages and low rates of integration (**f** = 1, **λ** = 10^−9^), we observe oscillatory coexistence of all populations. **B1-B2. Sputnik model:** cells with an integrated virophage cannot establish in the Sputnik mechanism. The Mavirus trajectories converge to a 4-dimensional limit cycle in state-space (shown as two nested trajectories), while the Sputnik trajectories converge to a 3-dimensional limit cycle. **A3-A4. Mavirus model:** at a higher integration rate (**λ** = 10^−4^), we observe replacement of naïve cells by cells with an integrated virophage. **B3-B4. Sputnik model:** increasing the integration rate leads to coexistence of naïve cells and cells with a provirophage. Parameters for model 1: **α**_**1**_ = 1, **α**_**2**_ = 0.9, **β**_**1**_ = 10^−7^, **β**_**2**_ = 10^−6^, **γ**_**1**_ = 1.2, **γ**_**2**_ = 2.6, **φ**_**1**_ = 100, **φ**_**2**_ = 1000, **f** = 1, **K** = 10^6^, **r**_**1**_ = 1, **r**_**2**_ = 0.8, **λ** = {10^−9^,10^−4^}. Parameters for model 2 are the same except for **β**_**2**_ = 10^−7^ and **k** = 8⋅10^−7^. Initial conditions: **C**_**x,0**_ = 10^5^, **G**_**0**_ = 10^5^, **V**_**0**_ = 10^5^.

**Fig 3:**
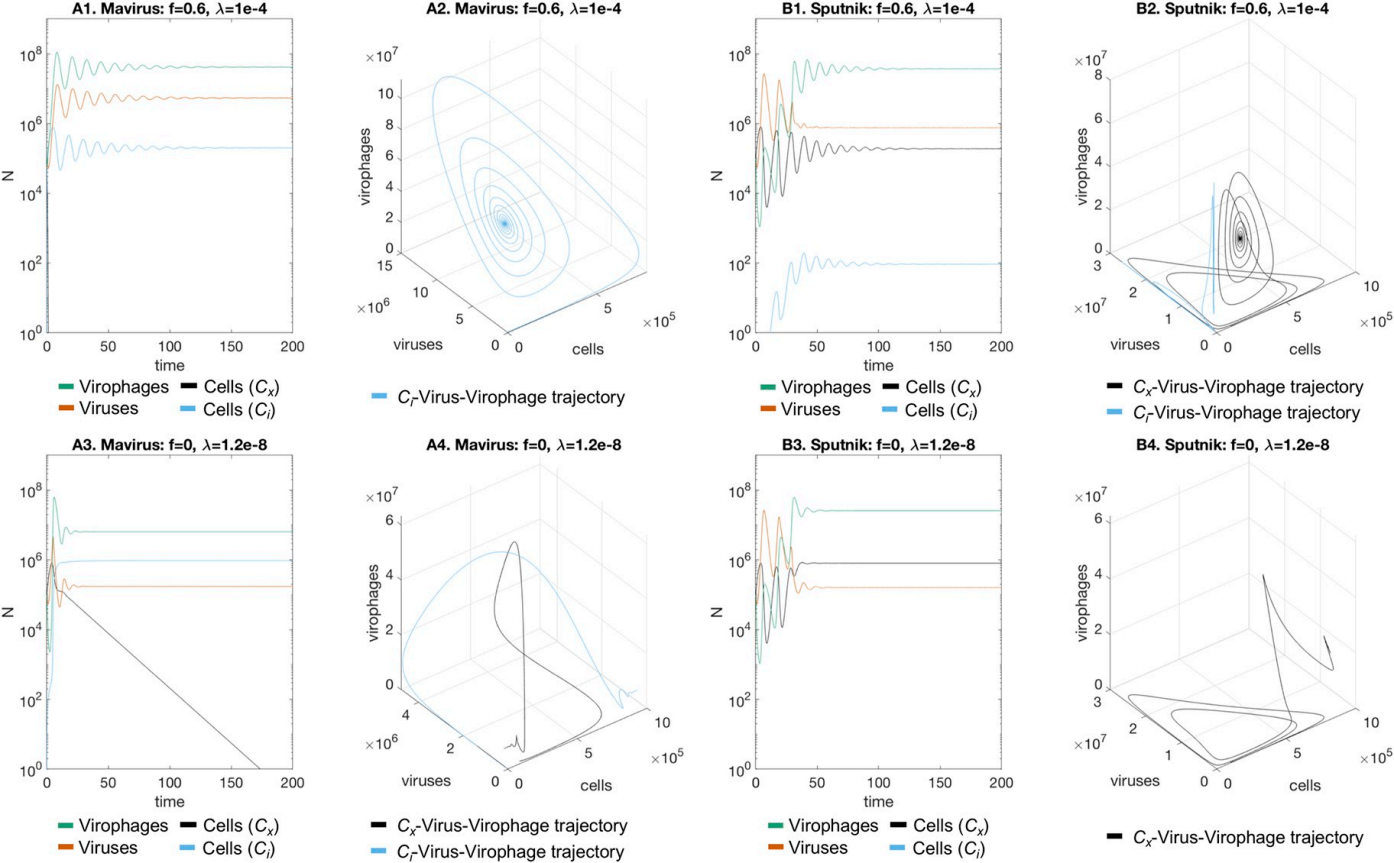
Dynamics of the Mavirus and Sputnik 1a models in the presence of virophage integration (inhibitory virophages). In the presence of inhibitory virophages (f = 0.6) all models converge to a point equilibrium. **A1-A2. Mavirus model:** at high integration rates (**λ** = 10^−4^), replacement of the population with cells carrying a provirophage is observed for the Mavirus model, trajectories form an inward spiral. **B1-B2. Sputnik model:** an integration rate of **λ** = 10^−4^ leads to coexistence of naïve cells and cells with a provirophage. **A3-A4. Mavirus model:** a lower integration rate (**λ** = 1.2 10^−8^) still leads to replacement. **B3-B4. Sputnik model:** a lower integration rate of **λ** = 1.2 10^−8^ leads to persistence of naïve cells. Parameters for model 1: **α**_**1**_ = 1, **α**_**2**_ = 0.9, **β**_**1**_ = 10^−7^, **β**_**2**_ = 10^−6^, **γ**_**1**_ = 1.2, **γ**_**2**_ = 2.6, **φ**_**1**_ = 100, **φ**_**2**_ = 1000, **f** = 0, **K** = 10^6^, **r**_**1**_ = 1, **r**_**2**_ = 0.8, **λ** = {1.2 10^−8^,10^−4^}. Parameters for model 2 are the same except for **β**_**2**_ = 10^−7^ and **k** = 8⋅10^−7^. Initial conditions: **C**_**x,0**_ = 10^5^, **G**_**0**_ = 10^5^, **V**_**0**_ = 10^5^.

Inclusion of integrating virophages affected dynamics of the system differently depending on whether virophages followed the Mavirus or Sputnik infection mechanisms. It is important to bear in mind that the parameter **λ** is interpreted differently across models: in the Mavirus model it is the rate of virophage integration, in the Sputnik model 1a it is the rate of abortive infection followed by virophage integration, and in the Sputnik model 1b it is the proportion of viruses carrying an integrated virophage. When virophages are neutral and the rate of integration is low (**f** = 1, **λ** = 10^−9^), we observed the appearance of cells with an integrated virophage which oscillated together with naïve cells, virophages and viruses in the Mavirus model (Fig 2A1-A2). In the Sputnik model, naïve cells oscillate with viruses and virophages when there is no inhibition and integration rate is low (**f** = 1, **λ** = 10^−9^) (Fig 2B1-B2). If we then increased the integration rate (**f** = 1, **λ** = 10^−4^), cells with an integrated virophage were observed in the Sputnik model (Fig 2B3-B4), while these cells replaced the naïve cell population in the Mavirus model (Fig 2A3-A4). We then considered a case with moderate inhibition (f = 0.6) and an integration rate **λ** = 10^−4^, where we observed stabilisation of the oscillations for both models with replacement in the Mavirus model (Fig 3A1-A2), and coexistence in the Sputnik model (Fig 3B1-B2). Under total virophage inhibition and low integration rate (**f** = 0, **λ** = 1.2⋅10^−8^), both models also showed damped oscillations converging to a point equilibrium, with replacement of naïve cells in the Mavirus model (Fig 3A3-A4), and stable coexistence in the Sputnik model (Fig 3B3-B4). We estimated the critical value of **f** (f*) at which the shift in dynamics occurs for both models (with **λ** = 10^−4^), by calculating the eigenvalues of the Jacobian matrix evaluated at the equilibrium solution (S1 and S2 Tables). This shift occurs at f* = 0.8073 for the Mavirus model, and at f* = 0.8303 for the Sputnik model, when the real part of the complex conjugate eigenvalues becomes zero. An example of the change in the state-space trajectories is shown in S2 and S3 Figs. We also examined the behaviour of the system when r_1_ = r_2_ (setting both equal to 1), which resulted in qualitatively similar dynamics as for the system where r_1_ = 0.8r_2_ (S4 and S5 Figs).

**Fig 4:**
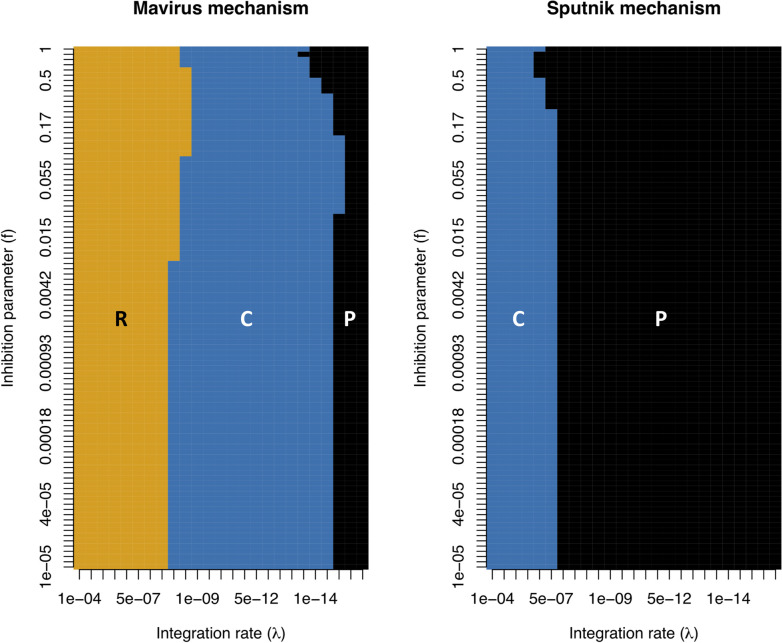
System outcomes depending on the virophage infection mechanism (log-log plot). We explored the outcome of the Mavirus and Sputnik models by varying the integration rates (**λ**) and the inhibition parameter (**f**), and mapping the dynamical outcomes. The degree of inhibition that has been measured experimentally seems to range from 1 (neutral) to 0.01 [13,14]. **Mavirus model (left)**: we observed three possible outcomes, fixation of cells with an integrated virophage (**R**: replacement, orange), coexistence of cells with and without an integrated virophage (**C**: coexistence, blue) and persistence of cells without a virophage integration (**P**: persistence, black). **Sputnik model (right)**: two outcomes were observed for the same parameter ranges, coexistence of cells with and without and integrated virophage (C, orange) and persistence of naïve cells (P, blue). Parameters for model 1: **α**_**1**_ = 1, **α**_**2**_ = 0.9, **β**_**1**_ = 10^−7^, **β**_**2**_ = 10^−6^, **γ**_**1**_ = 1.2, **γ**_**2**_ = 2.6, **φ**_**1**_ = 100, **φ**_**2**_ = 1000, **K** = 10^6^, **r**_**1**_ = 1, **r**_**2**_ = 0.8, **p** = 0. Parameters for model 2 are the same except for **β**_**2**_ = 10^−7^ and **k** = 8⋅10^−7^. Initial conditions: **C**_**x,0**_ = 10^5^, **G**_**0**_ = 10^5^, **V**_**0**_ = 10^5^.

We examined these effects on the system outcomes more systematically by sampling the inhibition parameter and the integration rate over multiple orders of magnitude (Fig 4). For both models, we observed that the main parameter determining system outcome is the integration rate, although it does interact to an extent with the inhibition parameter. In the Mavirus model, moderate rates of integration led to coexistence of naïve cells with cells carrying an integrated virophage (blue region, **C**), while higher integration rates led to replacement of naïve cells with cells carrying an integrated virophage (orange region, **R**). In contrast, the outcomes of the Sputnik model were dominated by naïve cells alone (black region, **P**), while coexistence of cell populations was only observed at high rates of integration; higher than those required to observe replacement in the Mavirus model (orange region, **R**).

### Integration of Sputnik into the virus genome

We also explored integration of Sputnik into the host virus genome since it has been observed experimentally [19]. In the absence of integrating virophages (λ* = 0), the system showed the same oscillatory dynamics with neutral virophages and damping oscillations under virophage inhibition, as described for the previous two models (S6 Fig). An integration frequency of 1/10,000 (λ* = 10^−4^) led to the appearance of viruses with an integrated virophage under no to moderate inhibition (**f** = {1, 0.7}; S7A1-A3 Fig). However, in the presence of total virophage inhibition (**f** = 0), viruses with an integrated virophage did not establish in the population (S7A4-A6 Fig). This makes intuitive sense since a virus carrying a totally inhibitory virophage would not be able to replicate.

### Agent-based model

We developed an agent-based model (ABM) in 3-dimensions to understand the impact of spatial effects in a stochastic simulation of cell-virus-virophage interactions. In particular, we were interested in understanding the effect of multicellularity on the system dynamics and how it interacted with virophage inhibition and programmed cell-death (PCD). For this, we considered 15 different scenarios in which populations of organisms with different cell numbers (single-, 2-, 4-, 8- and 16-celled organisms) were exposed to neutral virophages, inhibitory virophages or PCD. Our ABM considered a population of non-dividing cells in order to reduce the computational complexity of the simulations. We also provide the time-course results from simulations with a second model which allowed cell division for comparison (S11–S13 Figs).

### The effect of virophage inhibition, multicellularity and PCD

In the simulations we can see how the cell population was infected by viruses producing clouds of infection and bursts of virophages whenever they happened to coinfect a cell (Videos 1–15 in 10.6084/m9.figshare.19412066, S2 Text). An example time-course gives an idea of the underlying dynamics (S8–S10 Figs). In a population of single-celled organisms, the neutral virophage simulation produced the largest waves of viruses and the lowest numbers of cell survivors. Both virophage inhibition and PCD appeared to have a protective effect on the cell populations, but PCD was not protective when division of cells was considered (S11–S13 Figs). Increasing the number of cells per organism (which introduces spatial clustering of cells), also had a protective effect since the waves for viruses were lower and it increased the number of cell survivors compared to the single-celled case.

**Fig 5:**
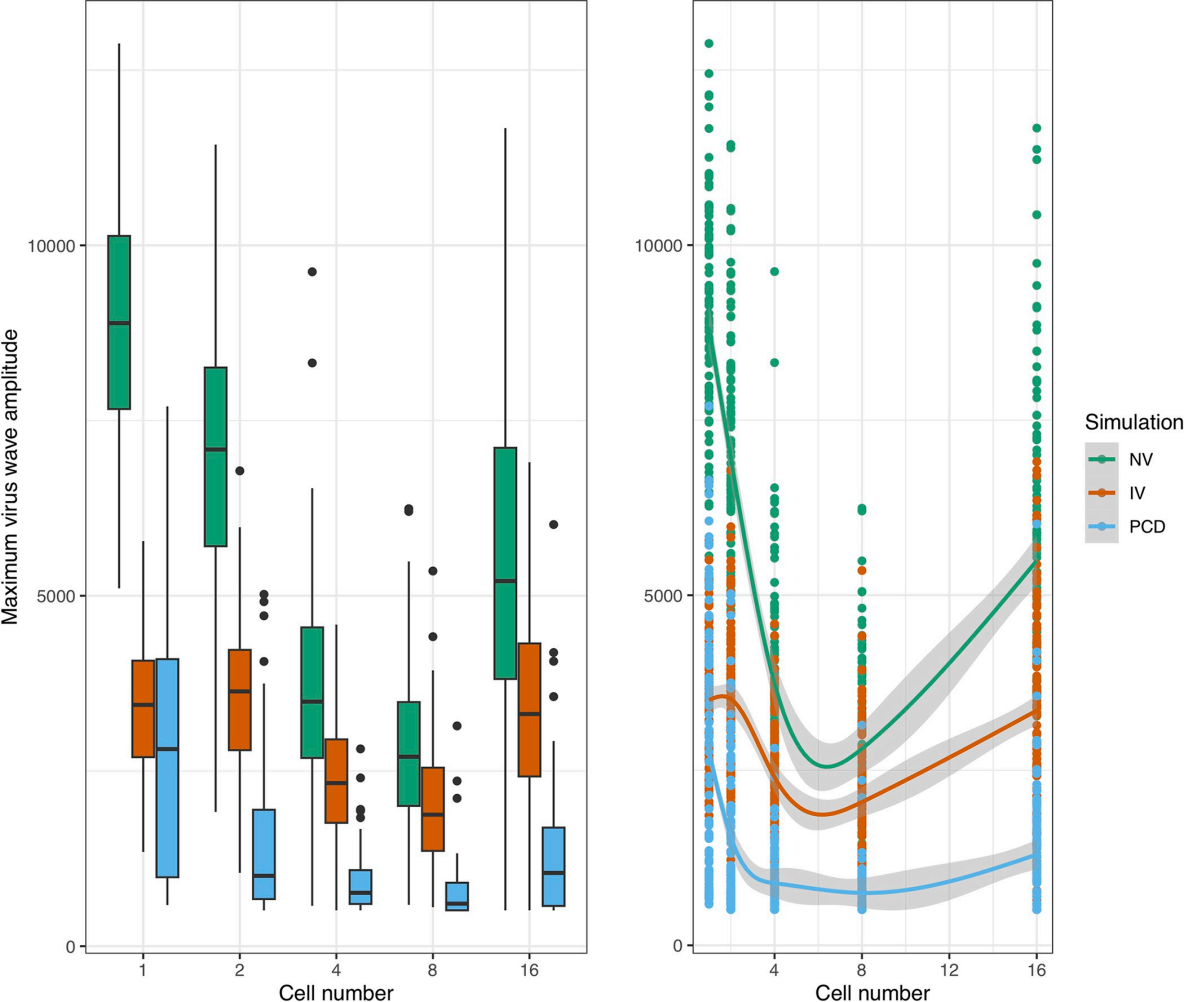
Maximum amplitude of the virus wave. The maximum amplitude of the virus wave decreases as a function of the number of cells and the simulation condition. Boxplots of the resulting distributions are shown to the left and the fitted GAMs are shown to the right. However, note the increase in the maximum wave amplitude across all simulations from 8- to 16-celled organisms. The three intercepts are significantly different and the smoothing terms statistically significant to the level of p < 10^−15^. The normalised root mean square error (RMSE) is 11% and the deviance explained is 72%. NV: neutral virophages, IV: inhibitory virophages, PCD: programmed cell-death.

**Fig 6:**
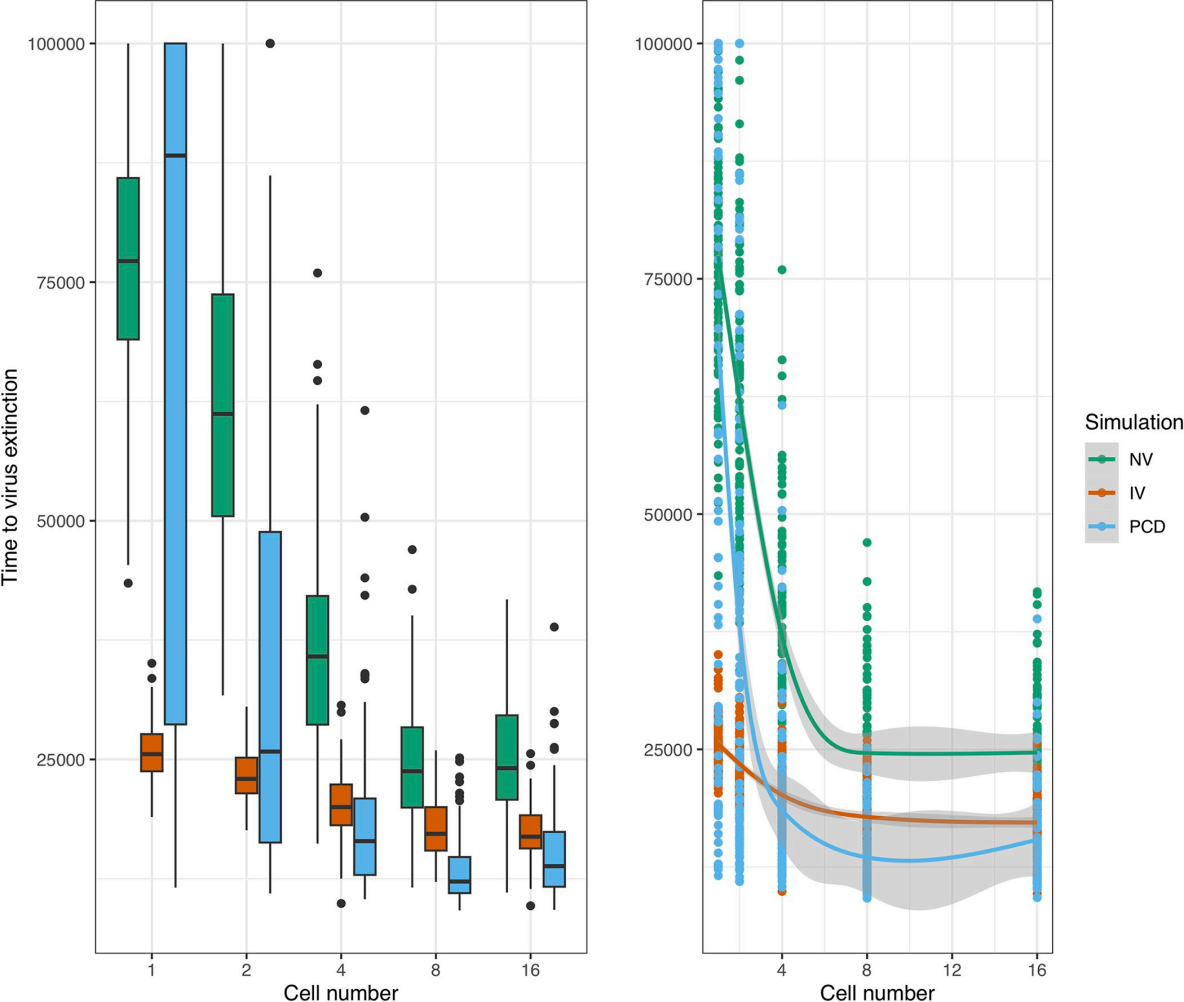
Time to virus extinction. The time to virus extinction also decreases as a function of the cell number and condition in the simulations. Boxplots of the resulting distributions are shown to the left and the fitted GAMs are shown to the right. For single-celled organisms, virophage inhibition was more effective than PCD, since it led to faster extinction of viruses. The simulations with neutral and inhibitory virophages appear to level-off with increasing cell number, while there was an increase in the time to virus extinction in the PCD simulation from 8- to 16-celled organisms (despite the variance being larger). The three intercepts are significantly different and the smoothing terms statistically significant for the neutral virophages and PCD to the level of p < 10^−15^. The smoothing term for the simulation with inhibitory virophages was significant to the level of p < 10^−5^. The normalised root mean squared error (RMSE) is 14.6% and the deviance explained is 69.3%. NV: neutral virophages, IV: inhibitory virophages, PCD: programmed cell-death.

**Fig 7:**
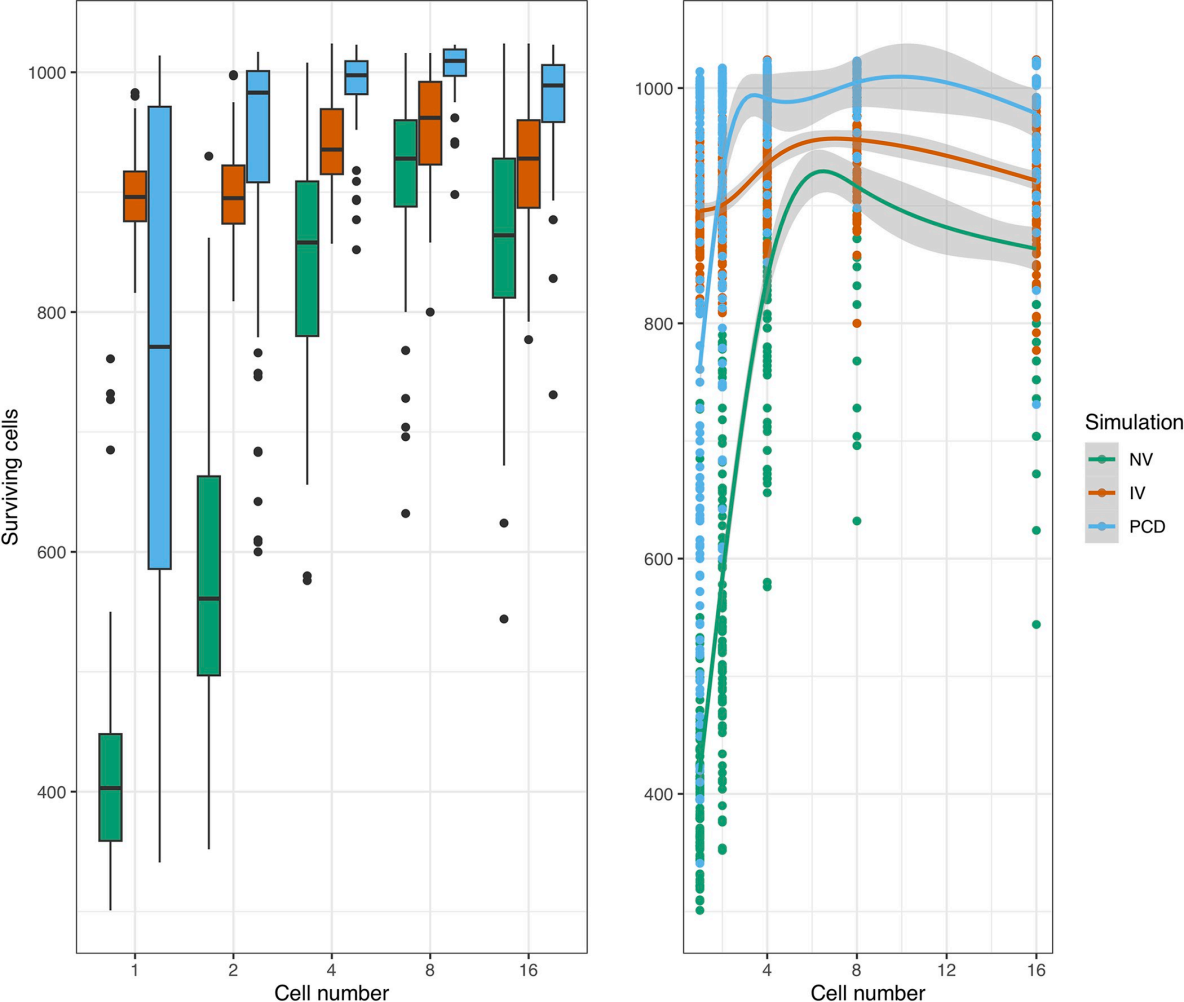
Number of surviving cells. The number of surviving cells at the end of the simulations increased as a function of the cell number and the simulation condition. Boxplots of the resulting distributions are shown to the left and the fitted GAMs are shown to the right. The highest levels of survival were observed for the PCD simulation, followed by virophage inhibition. The three intercepts are significantly different and the smoothing terms statistically significant for the neutral virophages and PCD to the level of p < 10^−15^. The smoothing term for the simulation with inhibitory virophages was significant to the level of p < 10^−5^. The normalised root mean squared error (RMSE) is 0.7% and the deviance explained is 76.8%. NV: neutral virophages, IV: inhibitory virophages, PCD: programmed cell-death.

To assess these effects systematically, we carried out 100 replicates of the stochastic simulations per condition and then analysed them using Generalised Additive Models (GAMs, DOI: 10.6084/m9.figshare.19412468). Compared to a population of single-celled organisms, increasing the cell number led to a general decrease in the maximum amplitude of the virus wave up to 8-celled organisms, although an increase was then observed from the 8- to 16-celled populations (Fig 5). The simulations with virophage inhibition had lower maximum amplitudes of the virus wave compared to neutral virophages, and the most effective condition was PCD (Fig 5). Multicellularity also lowered the time to virus extinction across conditions, and the protective effects of virophage inhibition and PCD were also observed (Fig 6). Finally, the number of surviving cells was highest for the PCD simulation, followed by the simulation with inhibitory virophages, and increased as a function of the number of cells per organism (Fig 7). However, when considering a model that allowed for cell reproduction, the most effective strategy was virophage inhibition, while PCD did not seem to offer protection (S11–S13 Figs).

## Discussion

Our findings reveal several key aspects of the natural history of virophages and the interactions with their viral and cellular hosts. We have shown that increasing the inhibition of virophages on virus replication can have a stabilising effect on the oscillatory dynamics. This is important since non-oscillatory stable systems are more robust to stochastic variations that can lead to collapse of the populations [32]. A previous model that did not consider the populations of viruses and virophages explicitly, suggested that increasing inhibition would be destabilising, leading to oscillatory dynamics and making the populations prone to extinction [32]. To reconcile this with the observed abundance and persistence of virophages in nature [23,33], the existence of a viral reservoir or meta-population dynamics were proposed [32]. However, our models show that the long-term stability of virophages and giant viruses in nature is possible without the need of external intervening factors, although it is likely that multiple factors may be at play. Our predictions could be tested by comparing the dynamics of a neutral virophage (e.g. Zamilon) and a strongly inhibitory virophage (e.g. Mavirus), measured by quantifying the amount of nucleic acids by a method such as qPCR. Adding neutral virophages to oscillating populations of cells and viruses is expected to result in oscillations, while adding a strongly inhibitory virophage to an oscillating population of cells and viruses is predicted to result in loss of the oscillatory dynamics.

Another important observation that emerged from our analyses is that integration of virophages into the cell or virus genomes is potentially determined by the virophage infection mechanism. Virophages that follow an independent-entry mechanism such as Mavirus would be expected to be more commonly found in the genomes of their eukaryotic hosts, while viruses that enter the cells as a complex would be harder to be found integrated in cells. These predictions are consistent with experimental observations: Mavirus and Mavirus-like virophages have been found in the genomes of *Cafeteria burkhardae* and *Bigelowiella natans* [13,18,34], while Sputnik and Sputnik-like virophages have so far been found integrated in the genomes of their virus hosts [17,19,20]. One important aspect about the natural history of the Sputnik system is that a virophage integration in a cell can only survive if it occurs together with an abortive viral infection, otherwise the cell is lysed and its descendants will not inherit the integration [17], thus making it harder for integrated virophages to be found.

In addition, we observed that when Sputnik virophages integrated into a high proportion of viral progeny (>1/10,000), viruses with integrated virophages could persist stably in the population. High rates of integration into the viral genome could indicate that Sputnik has evolved a greater affinity for integration into the virus compared to the host cell, probably as a consequence of its unique infection mechanism. Indeed, tyrosine recombinases do seem capable of integrating into the host cell genome, as shown by a *Polinton*-like virus that was discovered integrated in the genome of the cryptophyte alga *Guillardia theta* [35]. It would be interesting to test if changing the infection mechanisms of Mavirus and Sputnik leads to different integration dynamics as predicted by our models. For example, Mavirus and Sputnik may be genetically modified to use each other’s outer capsid proteins and host virus (i.e. Mavirus/APMV and Sputnik/CroV), and it could then be seen which integration pattern they follow.

Our analyses also indicate that virophage inhibition, virus-induced programmed cell-death (PCD) and the transition to multicellularity can be effective antiviral strategies in microbial eukaryotes. PCD has been classically studied in the context of complex multicellular organisms, where is has important functions in development and homeostasis [36], but PCD also occurs in microbes [37]. This gives rise to a paradox since the suicide of a single cell ends its reproductive potential. However, artificial life models have shown that kin selection is sufficient to explain the evolution of suicide in single-celled organisms [38]. In *Escherichia coli*, cell-suicide has been shown to be advantageous in the presence of lytic phage T4*rII* even when relatedness is low: the benefit to the closest kin greatly exceeds the cost to the infected cell since it will die anyway [39]. We did not observe a protective effect from PCD in simulations which allowed cells to actively reproduce, and therefore it seems that the benefit gained by PCD can be influenced by other demographic processes such as reproduction. In the simulations, we observed that a multicellular population of organisms gains protection against viral infection compared to a population of single-celled organisms. Our explanation for this spatial effect is that as long as viruses decay over time (or are lost to the environment), hosts that are more widely dispersed in space will be harder to reach. Therefore, it is more likely that the virus will decay if it has to traverse a longer diffusing distance, while in the single-celled case, it can use hosts as stepping-stones to travel through space more easily. Our results are consistent with previous modelling work that used a Markov process to show that cell-clustering and PCD are optimal strategies in the presence of high viral loads and imperfect immunity [40].

We have developed a number of exploratory models to gain insights into the dynamics of cell-virus-virophage systems. However, experimental follow-up will be necessary to fully understand this unique hyperparasitic system. In particular, we see as priorities measuring the integration rates of Mavirus and Sputnik virophages, in order to estimate the magnitude of the integration parameters in each case. It would be ideal if the population dynamics of several cell-virus-virophage systems could be assessed in a comparative framework, in order to maximise the chances of observing different dynamical outcomes. In the case of Sputnik, it will also be crucial to quantify integration into the virus genome and compare it to the frequency of integration into the cell genome. These experiments can provide key observations which would enable the development of more exact mathematical models to describe the system.

We have provided mechanistic insights into the interactions of virophages with their virus and cellular hosts, and at the same time explored antiviral defence strategies that are at the disposal of microbial eukaryotes. These results are relevant to the evolution of antiviral immunity in early eukaryotes and to the evolution of multicellularity. Indeed, there is evidence to support horizontal gene transfers between NCLDVs and proto-eukaryotes, which indicates these viral lineages already existed more than a billion years ago [25,27]. Virophages are also believed to be an ancient group of viruses, given their diversity, abundance and association with multiple NCLDV host families [23,33,41]. In light of this ancient arms-race, the early eukaryotes may have used inhibitory virophages to protect themselves against the onslaught from NCLDVs. The interaction with viruses may also have contributed to the initial transition to multicellularity. In this regard, it would be interesting to test if simple colonial forms of unicellular eukaryotes can evolve in the presence of a lytic virus, as they can do when exposed to a predator [42,43]. Other processes, such as the sharing of public goods, may have also played major roles in the evolution of multicellularity and PCD [38,44]. Since they do not appear to be mutually exclusive, it seems likely that the interaction with viruses and the sharing of public goods may have acted together to favour the appearance of multicellular eukaryotes on multiple occasions. The extent to which viruses may be a driving or reinforcing factor during these transitions can be explored in future work.

## Materials and methods

We used two main approaches to study the dynamic interactions between cells, viruses and virophages in the presence of virophage integration. Our first approach was to design and study the qualitative behaviour of coupled systems of Ordinary Differential Equations (ODEs). These systems considered the different virophage infection mechanisms and integration into the cell or host virus genomes. The second approach was to use an Agent-based model (ABM) which allowed us to consider spatial effects and analyse the effect of multicellularity on the dynamics of the system. The codes and scripts developed to implement these models are available on GitHub (https://github.com/josegabrielnb/virophage-dynamics).

### ODE models

To study the impact of different infection mechanisms and virophage integration, we developed three systems of coupled ODEs. Our systems of ODEs explicitly model the populations of cells, viruses and virophages, and assume that the system is well-mixed. The first system is based on the independent-entry mechanism of Mavirus and integration into the host cell genome. The second system describes the paired-entry mechanism of Sputnik (the virophage enters the cell in a complex with the virus), and integration into the cell genome. We assumed that integrated virophages do not impair viral replication when reactivated, an assumption which is not inconsistent with experimental observations [13,17]. This assumption is also interesting from a theoretical perspective, since it implies that any benefit gained by cells with an integrated virophage is indirect. However, the validity of this assumption could be tested directly from experiments at the individual-cell level to inform future modelling work. The third model is also based on the paired-entry mechanism but considers integration of Sputnik into the host virus genome, which has been observed experimentally [19].

### Mavirus model

**Fig 8:**
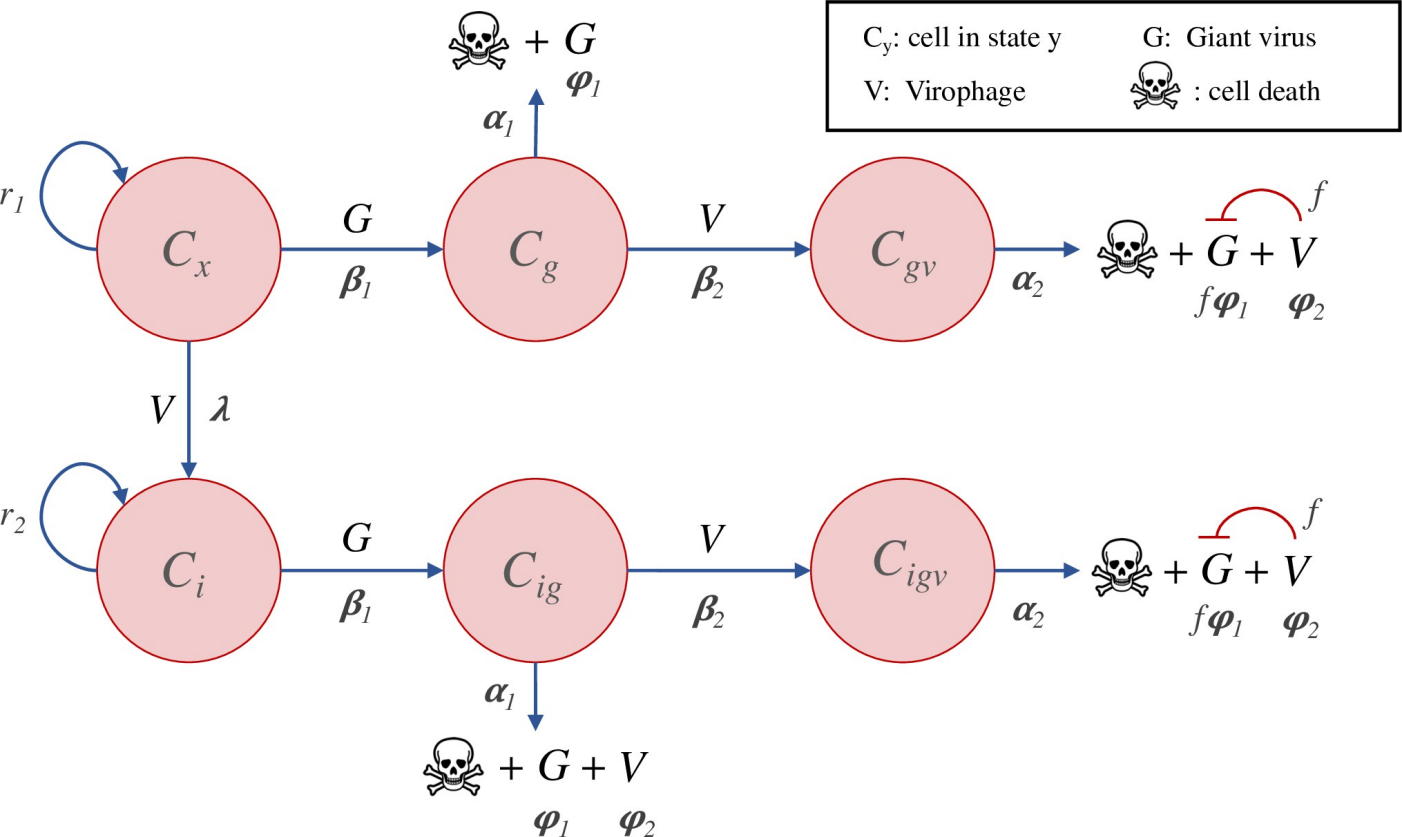
Model of virophage integration into the cell genome following the Mavirus mechanism. Cells with (**C**_**i**_) or without an integrated virophage (**C**_**x**_) can be infected by a virus. Infection of **C**_**x**_ cells by a virus results in lysis and release of viruses, while infection of **C**_**i**_ cells by a virus leads to virophage reactivation and release of viruses without inhibition. Cells infected by a virus (**C**_**g**_ or **C**_**ig**_) can also be infected by a virophage, which results in lysis, release of virophages and virus inhibition.

The Mavirus model assumes that the virophage can enter the cell independently of its host virus (Fig 8). Naïve cells (**C**_**x**_) can be infected by viruses (**G**) or virophages (**V**). Cells infected by a virus (**C**_**g**_), die at a certain rate and produce viral progeny, or they can be infected by a virophage. Cells that are infected by a virus and a virophage (**C**_**gv**_), produce viral progeny and release virophages when the cell dies. The amount of viral progeny produced is determined by the inhibition parameter (**f**), where **f** = 0 is total inhibition and **f** = 1 is no inhibition (neutral virophages). In the absence of a host virus, a virophage may infect and integrate into the genome of a naïve cell (**C**_**i**_). When this cell encounters a giant virus (**C**_**ig**_), the integrated virophage is reactivated but does not lead to virus inhibition. The virus-infected cell with a provirophage may also be infected by an exogenous virophage (**C**_**igv**_), which can have an inhibitory effect on viral replication. The model makes the simplifying assumption that virophages which infect a cell but which do not integrate are degraded. A similar assumption was made by Wodarz in model 1a [32]. This was done to arrive at a simpler model and to allow a more direct comparison with the Sputnik mechanism.

The Mavirus model is described by a system of 8 coupled ODEs and 13 parameters:

$$\left\{ \begin{array}{c} {C^{\prime }}_{x}=r_{1}C_{x}(1-C_{T}/K)-\beta_{1}C_{x}G-\lambda C_{x}V\\ {C^{\prime }}_{g}=\beta_{1}C_{x}G-\beta_{2}C_{g}V-\alpha_{1}C_{g}\\ {C^{\prime }}_{gv}=\beta_{2}C_{g}V-\alpha_{2}C_{gv}\\ {C^{\prime }}_{i}=r_{2}C_{i}(1-C_{T}/K)+{\lambda C}_{x}V-\beta_{1}C_{i}G\\ {C^{\prime }}_{ig}=\beta_{1}C_{i}G-\beta_{2}C_{ig}V-\alpha_{1}C_{ig}\\ {C^{\prime }}_{igv}=\beta_{2}C_{ig}V-\alpha_{2}C_{igv}\\ G^{\prime }=\alpha_{1}\varphi_{1}C_{g}+\alpha_{1}{f\varphi }_{1}C_{gv}+\alpha_{1}\varphi_{1}C_{ig}+\alpha_{2}f\varphi_{1}C_{igv}-\beta_{1}C_{x}G-\beta_{1}C_{i}G-\gamma_{1}G\\ V^{\prime }=\alpha_{2}\varphi_{2}C_{gv}+\alpha_{1}\varphi_{2}C_{ig}+\alpha_{2}\varphi_{2}C_{igv}-\beta_{2}C_{g}V-\beta_{2}C_{ig}V-\gamma_{2}V \end{array}\right.$$

Where **r**_**1**_ and **r**_**2**_ are the intrinsic growth rates of naïve cells and cells carrying a provirophage, **K** is the carrying capacity of cells in the population, **β**_**1**_ and **β**_**2**_ are the infection rates of viruses and virophages, **α**_**1**_ and **α**_**2**_ are the death rates of cells infected by a virus or a virus and virophage, **φ**_**1**_ and **φ**_**2**_ are the virus and virophage burst sizes, **γ**_**1**_ and **γ**_**2**_ are the decay rates for viruses and virophages, **λ** is the integration rate of virophages into the cell genome and **f** is the degree of virophage inhibition. In the models, CT refers to the total cell population.

### Sputnik model 1a: integration into the cell genome

**Fig 9:**
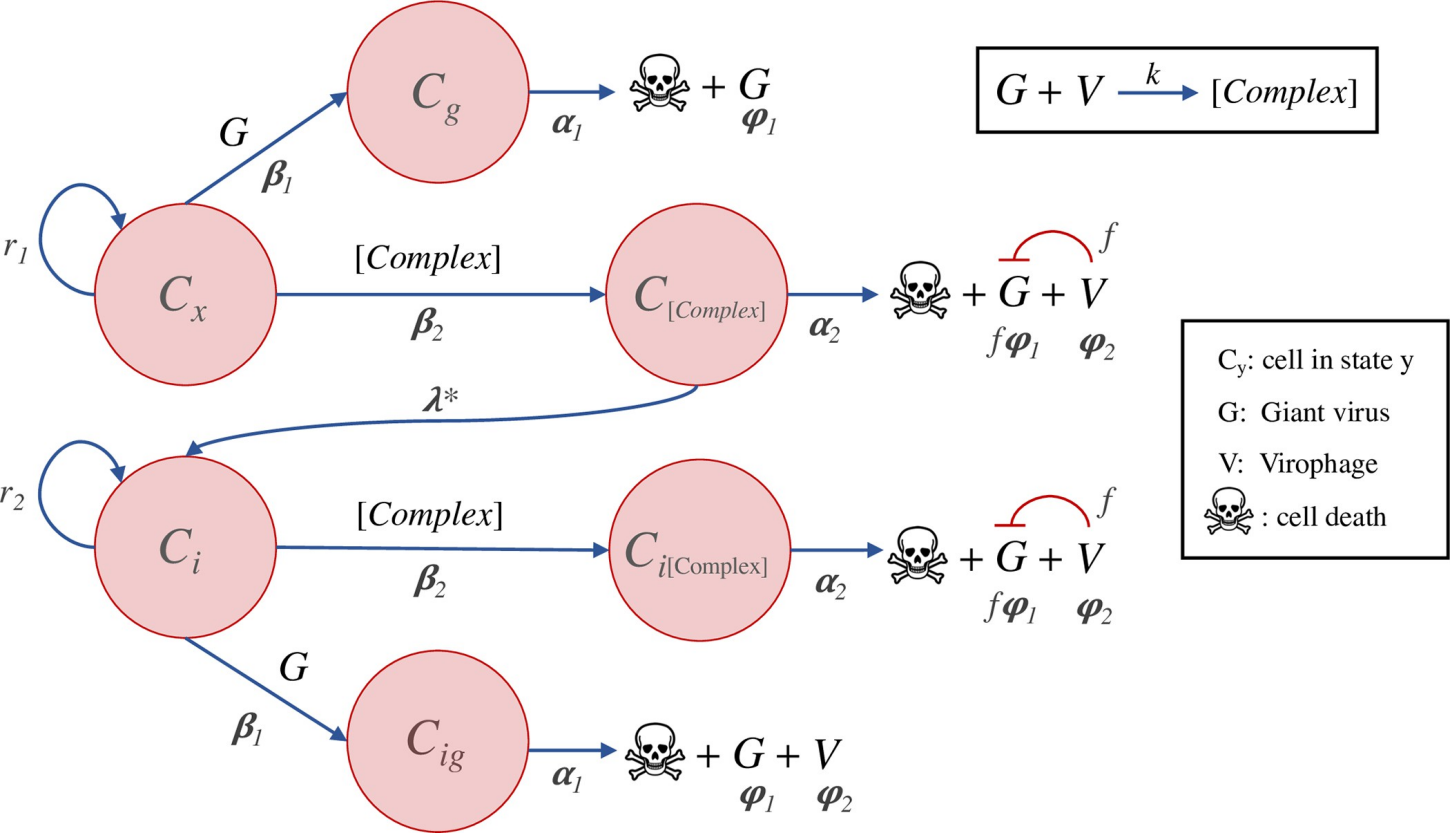
Model of virophage integration into the cell genome following the Sputnik mechanism. Cells with (**C**_**i**_) or without an integrated virophage (**C**_**x**_) can be infected by a virus (**G**) or by a virus-virophage complex ([Complex]). Infection of **C**_**x**_ cells by a virus results in lysis and virus release, while infection of **C**_**i**_ cells by a virus leads to virophage reactivation and release of viruses without inhibition. Infection of **C**_**x**_ or **C**_**i**_ cells by a complex, results in lysis, release of virophages and virus inhibition.

In contrast to Mavirus, Sputnik enters the cell by forming a complex with its host virus (Fig 9). In this model, naïve cells (**C**_**x**_) can be infected by a virus (**G**) or virus-virophage complex ([Complex]). Cells infected by the virus (**C**_**g**_) will die at a certain rate and produce viral progeny. Alternatively, cells may be infected by a complex (**C**_**[Complex]**_), release virophages and viral progeny at a level determined by the inhibition parameter (**f**). Since virophages can only enter the cell as a complex, the only way for a virophage to integrate into the cell genome is as a result of an abortive infection. Thus, the integration rate **λ** is a compound rate of integration and abortive infection. Cells with a virophage integration (**C**_**i**_), can be infected by a virus (leading to reactivation but no inhibition), or by a virus-virophage complex (leading to inhibition).

The Sputnik model with integration into the cell genome is described by a system of 9 coupled ODEs and 14 parameters:

$$\left\{ \begin{array}{c} {C\prime }_{x}=r_{1}C_{x}(1-C_{T}/K)-\beta_{1}C_{x}G-\beta_{2}C_{x}[Complex]\\ {C\prime }_{g}=\beta_{1}C_{x}G-\alpha_{1}C_{g}\\ {C\prime }_{\left[Complex\right]}=\beta_{2}C_{x}[Complex]-\lambda C_{\left[Complex\right]}-\alpha_{2}C_{\left[Complex\right]}\\ {C\prime }_{i}=r_{2}C_{i}(1-C_{T}/K)+\lambda C_{\left[Complex\right]}-\beta_{2}C_{i}[Complex]-\beta_{1}C_{i}G\\ {C\prime }_{ig}=\beta_{1}C_{i}G-\alpha_{1}C_{ig}\\ {C\prime }_{i[complex]}=\beta_{2}C_{i}[Complex]-\alpha_{2}C_{i[Complex]}\\ G^{\prime }=\alpha_{1}\varphi_{1}C_{g}+\alpha_{2}{f\varphi }_{1}C_{\left[Complex\right]}+\alpha_{1}\varphi_{1}C_{ig}+\alpha_{1}f\varphi_{1}C_{i[Complex]}-\beta_{1}C_{x}G-\beta_{1}C_{i}G-kGV-\gamma_{1}G+\gamma_{2}[Complex]\\ V^{\prime }=\alpha_{2}\varphi_{2}C_{\left[Complex\right]}+\alpha_{1}\varphi_{2}C_{ig}+\alpha_{2}\varphi_{2}C_{i[Complex]}-kGV-\gamma_{2}V+\gamma_{1}[Complex]\\ {\left[Complex\right]}^{\prime }=kGV-\beta_{2}C_{x}[Complex]-\beta_{2}C_{i}[Complex]-\gamma_{1}[Complex]-\gamma_{2}[Complex] \end{array}\right.$$

The parameters are the same as outlined for the Mavirus model, except for **β**_**2**_ which refers to the infection rate of the complex (assumed to be equal to **β**_**1**_), and **k** which is the rate of complex formation.

### Sputnik model 1b: integration into the virus genome

**Fig 10:**
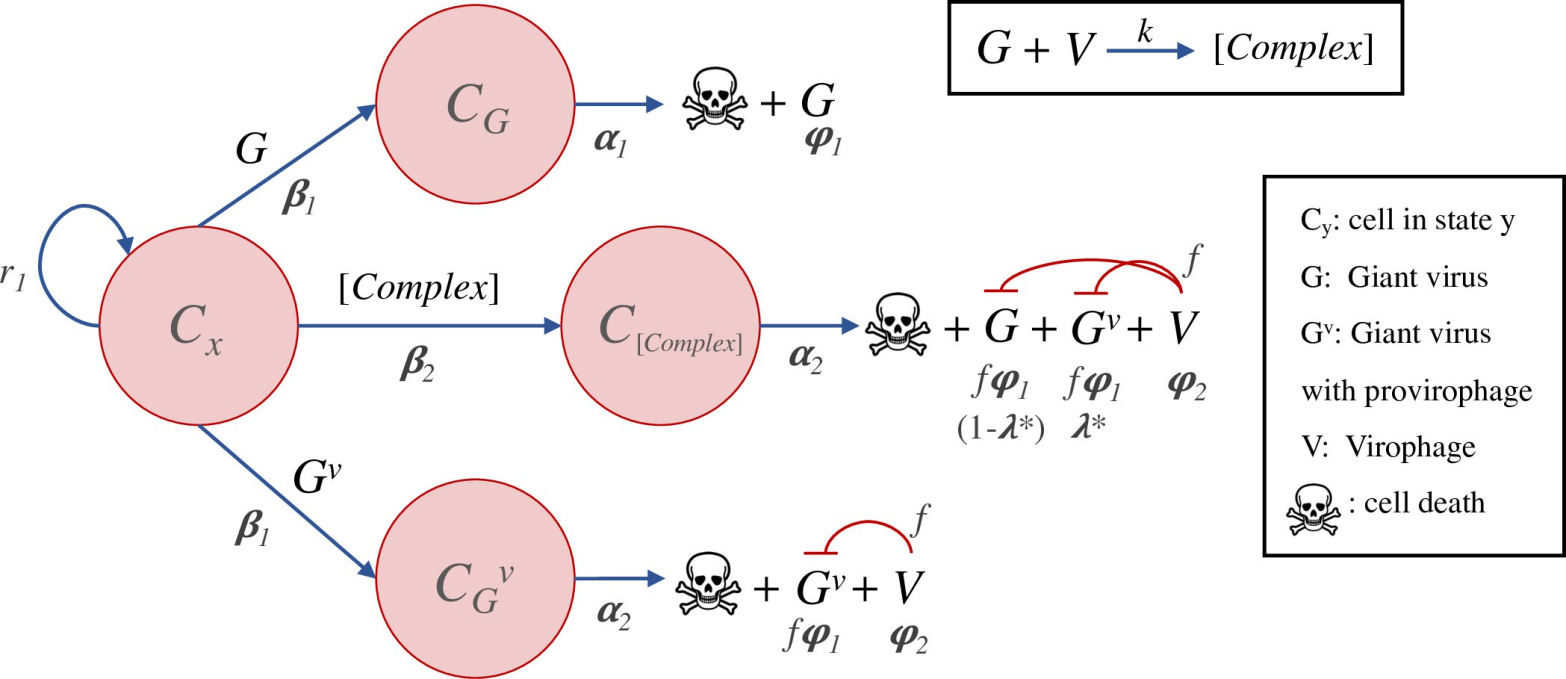
Model of virophage integration into the host virus genome following the Sputnik mechanism. Cells may be infected by a virus (**G**), a virus-virophage complex ([Complex]) or a virus carrying a provirophage (**G**_**v**_). During coinfection by a complex ([Complex]), a proportion λ* of the virus progeny incorporates a virophage into their genomes. Alternatively, when a cell is infected by a virus carrying a provirophage, the virus will produce progeny that also carry the integration, and at the same time will release virophages.

We examined a model in which Sputnik can integrate into the virus genome (Fig 10), given that Sputnik provirophages have been found in the genome of *Acanthamoeba polyphaga* Lentille virus [19]. In this model, naïve cells can be infected by a virus (**G**), by a complex ([Complex]) or a virus with an integrated virophage (**G**_**v**_). Infection by a virus carrying a provirophage results in production of the virus of the same genotype (**G**_**v**_) according to the inhibition parameter (**f**), and release of virophages. Alternatively, infection of a cell by a complex results in production of viral progeny according to **f**, release of virophages and a proportion of viruses with an integrated virophage (the proportion is λ*). A virus may also infect a naïve cell and replicate without inhibition.

The Sputnik model of integration into the virus genome is described by a system of 8 coupled ODEs and 13 parameters:

$$\left\{ \begin{array}{c} C_{x}^{\prime }=r_{1}C_{x}(1-C_{T}/K)-\beta_{1}C_{x}G-\beta_{1}C_{x}G_{v}-\beta_{2}C_{x}[Complex]\\ C_{G}^{\prime }=\beta_{1}C_{x}G-a_{1}C_{G}\\ C_{\left[Complex\right]}^{\prime }=\beta_{2}C_{x}[Complex]-a_{2}C_{i[Complex]}\\ C_{Gv}^{\prime }=\beta_{1}C_{x}G_{v}-a_{2}C_{Gv}\\ G^{\prime }=\alpha_{2}{f\varphi }_{1}(1-\lambda^{*})C_{\left[Complex\right]}+\alpha_{1}\varphi_{1}C_{G}-\beta_{1}C_{x}G-kGV-\gamma_{1}G+\gamma_{2}[Complex]\\ {G\prime }_{v}=\alpha_{2}{f\varphi }_{1}{\lambda^{*}C}_{i[Complex]}+\alpha_{2}\varphi_{1}C_{Gv}-\beta_{1}C_{x}G_{v}-\gamma_{1}G_{v}\\ {\left[Complex\right]}^{\prime }=kGV-\beta_{2}C_{x}[Complex]-\gamma_{1}[Complex]-\gamma_{2}[Complex]\\ V^{\prime }=\alpha_{2}\varphi_{2}C_{\left[Complex\right]}+\alpha_{2}\varphi_{2}C_{Gv}-kGV-\gamma_{2}V+\gamma_{1}[Complex] \end{array}\right.$$

The parameters have the same meanings as those we indicate for the model of Sputnik integration into the cell genome (Sputnik model 1a), except for λ* which is the proportion of viruses with an integrated virophage that are produced during infection with a complex.

### Numerical integration and choice of parameters

We integrated the models numerically in MATLAB 2020a using the ode45 solver for non-stiff differential equations [45,46]. We simulated the models under different scenarios by varying the integration rate of virophages (**λ**, range: [10^–^^16^, 10^–^^4^]) and the degree of virophage inhibition (**f**, range: [0 = total inhibition, 1 = no inhibition]). The values for the cell carrying capacity (**K**), cell intrinsic growth rate (**r**), virus burst size (**φ**_**1**_), virophage burst size (**φ**_**2**_) and the rate of complex formation (**k**) have been measured experimentally or estimated from biophysical first principles [47]. Therefore, we set our parameters to reflect the order of magnitude of these measurements. Cells carrying a provirophage incurred in a 20% cost of the intrinsic growth rate of naïve cells (**r**_**2**_ = 0.8**r**_**1**_). Our choices for the infection rates of viruses (**β**_**1**_) and virophages (**β**_**2**_), also comprised values in the order of magnitude of those that have been previously used to model this system [32,47]. We adjusted the death rate of hosts infected by a virus (**α**_**1**_) or by a virus and virophage (**α**_**2**_), and the decay rates of viruses (**γ**_**1**_) and virophages (**γ**_**2**_) so that they led to predator-prey oscillations in the presence of neutral virophages; this is our null hypothesis. We used this setup as a baseline scenario to examine the effect of varying the virophage integration rates (**λ**) and degrees of inhibition (**f**) on the system dynamics, which allowed comparison across the different models.

### Agent-based model

We developed an ABM in Julia [48], to study the effect of multicellularity, programmed cell-death (PCD) and virophage inhibition on the dynamics of the system. Cell, virus and virophage agents were modelled as mutable structures in Julia, with interactions in 3D-space. Cells changed their internal states as they interacted with viruses and virophages (analogous to ODE compartments). Viruses were assumed to follow an independent entry mechanism as in Mavirus. We did not consider dividing cells in this case since this considerably reduces the complexity of the simulations. Therefore, the model follows a single epidemic wave in a population of susceptible hosts.

We started the simulation with 1024 cells, 512 viruses and 2048 virophages. Each agent was assigned a random position in space by sampling a uniform distribution. During the simulation, each agent underwent Gaussian random walks. The size of the displacement for a particle depends on its diffusion coefficient, which according to the Einstein-Stokes equation is a function of the particle size [49]. Since virophages are the smallest particles in the system, we set an arbitrary displacement of 1 cell diameter per time step. The displacements of cells and viruses were expressed as a function of the virophage displacement (see S1 Text for derivation).

After every time step, we calculated the distances between cells and viruses, as well as the distance between cells and virophages. If the distance to a cell was less than 1 cell radius, we considered the cell to be infected. Cells infected by viruses died and released viral progeny after the incubation period. If the cell was infected by a virus and a virophage, the cell still died but released virophages and viral progeny according to the inhibition parameter. A cell only infected by a virophage could clear it after a certain number of time steps or alternatively, the virophage could integrate into the cell genome. A virus that infected a cell with an integrated virophage, killed the cell but released virophages and viruses without inhibition.

Multicellular organisms were modelled as cells that were attached to each other in a specified geometry and used the same random displacement vectors. We analysed the dynamics of systems with single-cell, 2-celled, 4-celled, 8-celled and 16-celled agents. Given that viruses and virophages are diffusing through space from the point of production, a cell in a multicellular organism is likely to become infected with viral/virophage progeny coming from an infected cell in the same organism, and to a certain degree this is analogous to cell-to-cell transmission. Virus induced cell-death was modelled by a constant probability of death per time step, given that a cell is infected by a virus. We analysed three simulation conditions for each population of cell agents: neutral virophages, inhibitory virophages and PCD.

We ran stochastic simulations in 100 replicates for each cell number group/condition and analysed the effects of simulations and multicellularity by fitting Generalised Additive Models (GAMs). We decided to use GAMs since we observed non-linear relationships in the data and these models allowed testing for multiple effects using smooth functions. Fitting was done using the mgcv package in R [50,51].

An additional model that allowed for cell reproduction was also implemented for comparison. The set up was the same as described above, but uninfected cells (cells without an active virus infection) could reproduce to a carrying capacity of 2000 individuals, by giving birth to unicellular progeny (development is not considered). Simulations were carried out for each of the 15 conditions, and the time-courses are presented in S11–S13 Figs.

## Supporting information

S1 Text Derivation of the displacements of cells and viruses relative to virophages. These results were used to set the displacements per time step in the ABM simulations.
(DOCX)

S2 Text Description of video files. (DOCX)

S1 Table Shift in the sign of the real part of the complex conjugate eigenvalues calculated for the Mavirus model (λ = 1e-4). First, the equilibrium point of the system was calculated (solution with positive number of cells, viruses and virophages), and then the eigenvalues were calculated from the Jacobian matrix of the system evaluated at this point. The critical value of f* (where the real part becomes zero) is at 0.8073 (asterisk).
(DOCX)

S2 Table Shift in the sign of the real part of the complex conjugate eigenvalues calculated for the Sputnik model (λ = 1e-4). First, the equilibrium point of the system was calculated (solution with positive number of cells, viruses and virophages), and then the eigenvalues were calculated from the Jacobian matrix of the system evaluated at this point. The critical value of f* (where the real part becomes zero) is at 0.8303.
(DOCX)

S1 Fig Dynamics of the Mavirus and Sputnik 1a models in the absence of viruses or virophages. **A1-A2, B1-B2.** In the absence of viruses, cells in the Mavirus and Sputnik models simply grow to carrying capacity. **A3-A4, B3-B4.** When viruses are added, predator-prey-like oscillations are observed for both models; the oscillations produce a 2D limit cycle in state space. Parameters for models: **α**_**1**_ = 1, **β**_**1**_ = 10^−7^, **γ**_**1**_ = 1.2, **φ**_**1**_ = 100, **K** = 10^6^, **r**_**1**_ = 1. Initial conditions (**A1-A2**, **B1-B2**): **C**_**x,0**_ = 10^5^, **G**_**0**_ = 0, **V**_**0**_ = 0. Initial conditions (**A3-A4**, **B3-B4**): **C**_**x,0**_ = 10^5^, **G**_**0**_ = 10^5^, **V**_**0**_ = 0.
(TIF)

S2 Fig Emergence of a limit cycle attractor in the Mavirus model. A1) When the inhibition parameter (**f**) is below the critical value of 0.8073, trajectories converge to a point equilibrium. **A2**) When f is increased over the critical value, trajectories converge to a limit cycle.
(TIF)

S3 Fig Emergence of a limit cycle attractor in the Sputnik model. A1) When the inhibition parameter (**f**) is below the critical value of 0.8303, trajectories converge to a point equilibrium. A2) When f is increased over the critical value, trajectories converge to a limit cycle.
(TIF)

S4 Fig Dynamics of the Mavirus and Sputnik 1a models in the presence of integration by neutral virophages (f = 1, r1 = 1, r2 = 1). **A.** In the Mavirus model, cells with a provirophage become fixed at low (**A1-A2**) and high (**A3-A4**) integration rates. **B.** In the Sputnik model, naïve cells are observed for low integration (**B1-B2**), while coexistence is observed at a higher integration rate (**B3-B4**). Parameters for model 1: **α**_**1**_ = 1, **α**_**2**_ = 0.9, **β**_**1**_ = 10^−7^, **β**_**2**_ = 10^−6^, **γ**_**1**_ = 1.2, **γ**_**2**_ = 2.6, **λ** = {1e-9, 1e-4}, **φ**_**1**_ = 100, **φ**_**2**_ = 1000, **f** = 1, **K** = 10^6^, **r**_**1**_ = 1, **r**_**2**_ = 1. Parameters for model 2 are the same except for **β**_**2**_ = 10^−7^ and **k** = 8⋅10^−7^. Initial conditions: **C**_**x,0**_ = 10^5^, **G**_**0**_ = 10^5^, **V**_**0**_ = 10^5^.
(TIF)

S5 Fig Dynamics of the Mavirus and Sputnik 1a models in the presence of integration by inhibitory virophages (f = 0, r1 = 1, r2 = 1). **A.** In the Mavirus model, cells with a provirophage become fixed at low (**A1-A2**) and high (**A3-A4**) integration rates. **B.** In the Sputnik model, naïve cells are observed for low integration (**B1-B2**), while coexistence is observed at a higher integration rate (**B3-B4**). Parameters for model 1: **α**_**1**_ = 1, **α**_**2**_ = 0.9, **β**_**1**_ = 10^−7^, **β**_**2**_ = 10^−6^, **γ**_**1**_ = 1.2, **γ**_**2**_ = 2.6, **λ** = {1.2e-8, 1e-4}, **φ**_**1**_ = 100, **φ**_**2**_ = 1000, **f** = 0, **K** = 10^6^, **r**_**1**_ = 1, **r**_**2**_ = 1. Parameters for model 2 are the same except for **β**_**2**_ = 10^−7^ and **k** = 8⋅10^−7^. Initial conditions: **C**_**x,0**_ = 10^5^, **G**_**0**_ = 10^5^, **V**_**0**_ = 10^5^.
(TIF)

S6 Fig Dynamics of the Sputnik 1b model in the absence of integration (λ* = 0). **A1.** Neutral virophages lead to a stable oscillatory regime. **A2-A3.** Virophage inhibition **f** = {0.7, 0} leads to loss of oscillations and stabilisation of the dynamics. Model parameters: **α**_**1**_ = 1, **α**_**2**_ = 0.9, **β**_**1**_ = 10^−7^, **β**_**2**_ = 10^−7^, **γ**_**1**_ = 1.2, **γ**_**2**_ = 2.6, **φ**_**1**_ = 100, **φ**_**2**_ = 1000, f = {1, 0.7, 0}, **K** = 10^6^, **r**_**1**_ = 1, **r**_**2**_ = 0.8, λ* = 0.
(TIF)

S7 Fig Dynamics of the Sputnik 1b model with integration into the host virus genome. Virophage inhibition leads to stabilisation of the dynamics. **A1-A3.** In the presence of moderate integration (λ* = 10^−4^), viruses with an integrated virophage can establish in the population and are maintained as a polymorphism (**f** = {1, 0.7}), unless there is total virophage inhibition (**f** = 0). **A4-A6.** In the presence of high integration (λ* = 0.2), the original virus population is replaced by viruses carrying an integrated virophage (**f** = {1, 0.7}), except when inhibition is total (**f** = 0). Model parameters: **α**_**1**_ = 1, **α**_**2**_ = 0.9, **β**_**1**_ = 10^−7^, **β**_**2**_ = 10^−7^, **γ**_**1**_ = 1.2, **γ**_**2**_ = 2.6, **φ**_**1**_ = 100, **φ**_**2**_ = 1000, **f** = {1, 0.7, 0}, **K** = 10^6^, **r**_**1**_ = 1, **r**_**2**_ = 0.8, **λ** = {10^−4^, 0.2}.
(TIF)

S8 Fig Time-course of the population of viruses during an ABM simulation. The total virus population during a stochastic simulation is shown as a function of time and grouped by the number of cells per organism and simulation. The highest virus wave is observed in the population of single-celled organisms in the presence of neutral virophages. By comparison, a simulation with inhibitory virophages and PCD show lower maxima of the virus waves. The effect of multicellularity can also be observed in the lower virus waves from the single-celled to 8-celled cases. Random seed = 1234.
(TIF)

S9 Fig Time-course of the population of virophages during an ABM simulation. The total virophage population during a stochastic simulation is plotted as a function of time and grouped by the number of cells per organism and simulation. We observe the same pattern as for the virus population, where the highest virophage numbers were attained in the simulation with neutral virophages and lower for inhibitory virophages and PCD. In contrast to viruses, virophages attain much larger population sizes. Multicellularity also has an effect on reducing the maximum size of the virophage waves. Random seed = 1234.
(TIF)

S10 Fig Time-course of the number of surviving cells during an ABM simulation. The total number of cells during a stochastic simulation is shown as a function of time and grouped by the number of cells per organism and simulation. We can observe a greater survival of cells in simulations with PCD and inhibitory virophages, while the lowest survival occurred in the simulations with neutral virophages. Grouping cells together in space also provided an advantage since the survival of cells increases with increasing number of cells per organism. Random seed = 1234.
(TIF)

S11 Fig Time-course of the population of viruses during an ABM simulation (with cell division). The total virus population during a stochastic simulation is shown as a function of time and grouped by the number of cells per organism and simulation. The highest virus wave is observed in the population of single-celled organisms in the presence of neutral virophages. The simulation with inhibitory virophages show lower maxima of the virus waves. The effect of multicellularity can also be observed in the lower virus waves from the single-celled to 8-celled cases. Random seed = 1234.
(TIF)

S12 Fig Time-course of the population of virophages during an ABM simulation (with cell division). The total virophage population during a stochastic simulation is plotted as a function of time and grouped by the number of cells per organism and simulation. We observe the same pattern as for the virus population, where the highest virophage numbers were attained in the simulation with neutral virophages and lower for inhibitory virophages. In contrast to viruses, virophages attain much larger population sizes. Multicellularity also has an effect on reducing the maximum size of the virophage waves. Random seed = 1234.
(TIF)

S13 Fig Time-course of the number of surviving cells during an ABM simulation (with cell division). The total number of cells during a stochastic simulation is shown as a function of time and grouped by the number of cells per organism and simulation. We can observe a greater survival of cells in simulations with inhibitory virophages. Grouping cells together also provided an advantage since the survival of cells increases with increasing number of cells per organism across simulations. Random seed = 1234.
(TIF)
