# Supplementary material for: Ecological and evolutionary dynamics of cell-virus-virophage systems
Source: PLoS Comput Biol. 2024 Feb 20;20(2):e1010925. doi: 10.1371/journal.pcbi.1010925 (PMC10906902; doi:10.1371/journal.pcbi.1010925)
Supplement: S1 Text — These results were used to set the displacements per time step in the ABM simulations. (DOCX) [file pcbi.1010925.s001.docx]

**S1 Text. Derivation of the displacements of cells and viruses relative to virophages**. These results were used to set the displacements per time step in the ABM simulations.

The Mean Squared Displacement (MSD) is defined as the deviation in the position of a particle with respect to a reference position with time. In statistical mechanics, it is expressed as an ensemble average:

$$\left( 1 \right)\mathrm{MSD}= \frac{1}{N} \sum_{i=0}^{N} \left| x_{(t)}^{i}-x_{(t=0)}^{i} \right|^{2}$$

Since we will consider a single particle moving from the origin, we can set N = 1 and *x^i^* at time zero equal to zero. Thus, we have the following expression for the MSD:

$$\left( 2 \right)\mathrm{MSD}= {{(x}_{(t)}^{i})}^{2}$$

For a random walk in *n* dimensions the MSD is equal to:

$$\left( 3 \right)\mathrm{MSD}=2nDt$$

where *D* is the diffusion coefficient for the particle and *t* is time. Since the particles are moving in 3 spatial-dimensions we then have:

$$\left( 4 \right)\mathrm{MSD}=6Dt$$

Now we use the Einstein-Stokes equation for the diffusion coefficient of spherical particles at low Reynolds numbers:

$$\left( 5 \right) D= \frac{k_{b}T}{6\pi\eta r}$$

where k_b_ is Boltzmann’s constant, T is the temperature in Kelvin, η is the viscosity of the fluid and r is the radius of a suspended particle.

To relate the displacements to the radius of the particle we substitute (5) in (4), giving:

$$\left( 6 \right)\mathrm{MSD}= \frac{k_{b}Tt}{\pi\eta r}$$

To find the mean displacement we solve for *x* in equation number (2):

$$\left( 7 \right) x= \sqrt{\mathrm{MSD}}$$

Now we are interested in finding the mean displacement of the virus with respect to the virophage:

$$\left( 8 \right) \frac{x_{virus}}{x_{virophage}}= \sqrt{\frac{\frac{k_{b}Tt}{\pi\eta r_{virus}}}{\frac{k_{b}Tt}{\pi\eta r_{virophage}}}}$$

$$\left( 9 \right) x_{virus}= x_{virophage}\sqrt{\frac{r_{virophage}}{r_{virus}}}$$

Similarly, the magnitude of the mean displacement of a cell relative to the virophage will be:

$$\left( 10 \right) x_{cell}= x_{virophage}\sqrt{\frac{r_{virophage}}{r_{cell}}}$$
