# Supplementary material for: Ecological and evolutionary dynamics of cell-virus-virophage systems
Source: PLoS Comput Biol. 2024 Feb 20;20(2):e1010925. doi: 10.1371/journal.pcbi.1010925 (PMC10906902; doi:10.1371/journal.pcbi.1010925)
Supplement: S2 Text — (DOCX) [file pcbi.1010925.s002.docx]

**Video 1 in** [10.6084/m9.figshare.19412066](https://figshare.com/s/b88b9d60f5ba24e3adbc)**. Simulation of a population of single-celled organisms interacting with viruses and neutral virophages.** The simulation was started with 1024 cells, 512 viruses and 2048 virophages. Cells are represented as blue, viruses are red and virophages are green. The axes are in micrometres. Random seed = 1234.

**Video 2 in** [10.6084/m9.figshare.19412066](https://figshare.com/s/b88b9d60f5ba24e3adbc)**. Simulation of a population of single-celled organisms interacting with viruses and inhibitory virophages.** The simulation was started with 1024 cells, 512 viruses and 2048 virophages. Cells are represented as blue, viruses are red and virophages are green. The axes are in micrometres. Random seed = 1234.

**Video 3 in** [10.6084/m9.figshare.19412066](https://figshare.com/s/b88b9d60f5ba24e3adbc)**. Simulation of a population of single-celled organisms, which can undergo virus-induced PCD, interacting with viruses and neutral virophages.** The simulation was started with 1024 cells, 512 viruses and 2048 virophages. Cells are represented as blue, viruses are red and virophages are green. The axes are in micrometres. Random seed = 1234.

**Video 4 in** [10.6084/m9.figshare.19412066](https://figshare.com/s/b88b9d60f5ba24e3adbc)**. Simulation of a population of 2-celled organisms interacting with viruses and neutral virophages.** The simulation was started with 1024 cells, 512 viruses and 2048 virophages. Cells are represented as blue, viruses are red and virophages are green. The axes are in micrometres. Random seed = 1234.

**Video 5 in** [10.6084/m9.figshare.19412066](https://figshare.com/s/b88b9d60f5ba24e3adbc)**. Simulation of a population of 2-celled organisms interacting with viruses and inhibitory virophages.** The simulation was started with 1024 cells, 512 viruses and 2048 virophages. Cells are represented as blue, viruses are red and virophages are green. The axes are in micrometres. Random seed = 1234.

**Video 6 in** [10.6084/m9.figshare.19412066](https://figshare.com/s/b88b9d60f5ba24e3adbc)**. Simulation of a population of 2-celled organisms, which can undergo virus-induced PCD, interacting with viruses and neutral virophages.** The simulation was started with 1024 cells, 512 viruses and 2048 virophages. Cells are represented as blue, viruses are red and virophages are green. The axes are in micrometres. Random seed = 1234.

**Video 7 in** [10.6084/m9.figshare.19412066](https://figshare.com/s/b88b9d60f5ba24e3adbc)**. Simulation of a population of 4-celled organisms interacting with viruses and neutral virophages.** The simulation was started with 1024 cells, 512 viruses and 2048 virophages. Cells are represented as blue, viruses are red and virophages are green. The axes are in micrometres. Random seed = 1234.

**Video 8 in** [10.6084/m9.figshare.19412066](https://figshare.com/s/b88b9d60f5ba24e3adbc)**. Simulation of a population of 4-celled organisms interacting with viruses and inhibitory virophages.** The simulation was started with 1024 cells, 512 viruses and 2048 virophages. Cells are represented as blue, viruses are red and virophages are green. The axes are in micrometres. Random seed = 1234.

**Video 9 in** [10.6084/m9.figshare.19412066](https://figshare.com/s/b88b9d60f5ba24e3adbc)**. Simulation of a population of 4-celled organisms, which can undergo virus-induced PCD, interacting with viruses and neutral virophages.** The simulation was started with 1024 cells, 512 viruses and 2048 virophages. Cells are represented as blue, viruses are red and virophages are green. The axes are in micrometres. Random seed = 1234.

**Video 10 in** [10.6084/m9.figshare.19412066](https://figshare.com/s/b88b9d60f5ba24e3adbc)**. Simulation of a population of 8-celled organisms interacting with viruses and neutral virophages.** The simulation was started with 1024 cells, 512 viruses and 2048 virophages. Cells are represented as blue, viruses are red and virophages are green. The axes are in micrometres. Random seed = 1234.

**Video 11 in** [10.6084/m9.figshare.19412066](https://figshare.com/s/b88b9d60f5ba24e3adbc)**. Simulation of a population of 8-celled organisms interacting with viruses and inhibitory virophages.** The simulation was started with 1024 cells, 512 viruses and 2048 virophages. Cells are represented as blue, viruses are red and virophages are green. The axes are in micrometres. Random seed = 1234.

**Video 12 in** [10.6084/m9.figshare.19412066](https://figshare.com/s/b88b9d60f5ba24e3adbc)**. Simulation of a population of 8-celled organisms, which can undergo virus-induced PCD, interacting with viruses and neutral virophages.** The simulation was started with 1024 cells, 512 viruses and 2048 virophages. Cells are represented as blue, viruses are red and virophages are green. The axes are in micrometres. Random seed = 1234.

**Video 13 in** [10.6084/m9.figshare.19412066](https://figshare.com/s/b88b9d60f5ba24e3adbc)**. Simulation of a population of 16-celled organisms interacting with viruses and neutral virophages.** The simulation was started with 1024 cells, 512 viruses and 2048 virophages. Cells are represented as blue, viruses are red and virophages are green. The axes are in micrometres. Random seed = 1234.

**Video 14 in** [10.6084/m9.figshare.19412066](https://figshare.com/s/b88b9d60f5ba24e3adbc)**. Simulation of a population of 16-celled organisms interacting with viruses and inhibitory virophages.** The simulation was started with 1024 cells, 512 viruses and 2048 virophages. Cells are represented as blue, viruses are red and virophages are green. The axes are in micrometres. Random seed = 1234.

**Video 15 in** [10.6084/m9.figshare.19412066](https://figshare.com/s/b88b9d60f5ba24e3adbc)**. Simulation of a population of 16-celled organisms, which can undergo virus-induced PCD, interacting with viruses and neutral virophages.** The simulation was started with 1024 cells, 512 viruses and 2048 virophages. Cells are represented as blue, viruses are red and virophages are green. The axes are in micrometres. Random seed = 1234.
