# Supplementary material for: Ecological and evolutionary dynamics of cell-virus-virophage systems
Source: PLoS Comput Biol. 2024 Feb 20;20(2):e1010925. doi: 10.1371/journal.pcbi.1010925 (PMC10906902; doi:10.1371/journal.pcbi.1010925)
Supplement: S1 Table — First, the equilibrium point of the system was calculated (solution with positive number of cells, viruses and virophages), and then the eigenvalues were calculated from the Jacobian matrix of the system evaluated at this point. The critical value of f* (where the real part becomes zero) is at 0.8073 (asterisk). (DOCX) [file pcbi.1010925.s003.docx]

**S1 Table. Shift in the sign of the real part of the complex conjugate eigenvalues calculated for the Mavirus model (**λ = 1e-4**).** First, the equilibrium point of the system was calculated (solution with positive number of cells, viruses and virophages), and then the eigenvalues were calculated from the Jacobian matrix of the system evaluated at this point. The critical value of **f*** (where the real part becomes zero) is at 0.8073 (asterisk).

| **Inhibition parameter (f)** | **Complex eigenvalues** |
| --- | --- |
| 0.8000 | -0.0008 ± 0.6*i* |
| 0.8050 | -0.0003 ± 0.6*i* |
| ***0.8073** | **± 0.6*i*** |
| 0.8075 | +0.00002 ± 0.6*i* |
| 0.8100 | +0.0003 ± 0.6*i* |
| 0.8200 | +0.001 ± 0.6*i* |
| 0.8500 | +0.004 ± 0.6*i* |
