# Supplementary figures and images for: Ecological and evolutionary dynamics of cell-virus-virophage systems
Source: PLoS Comput Biol. 2024 Feb 20;20(2):e1010925. doi: 10.1371/journal.pcbi.1010925 (PMC10906902; doi:10.1371/journal.pcbi.1010925)

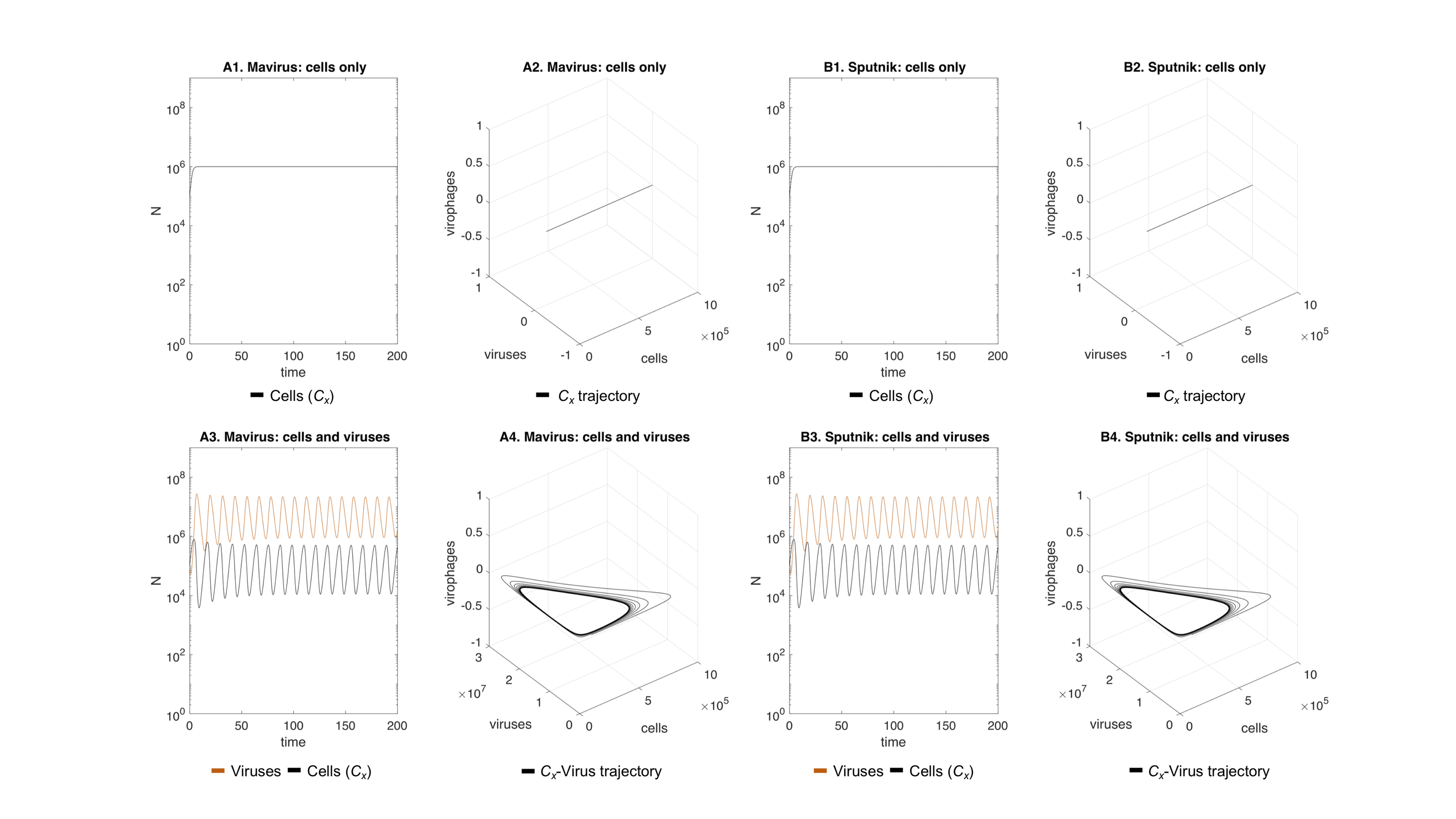

Supplement: S1 Fig — A1-A2, B1-B2. In the absence of viruses, cells in the Mavirus and Sputnik models simply grow to carrying capacity. A3-A4, B3-B4. When viruses are added, predator-prey-like oscillations are observed for both models; the oscillations produce a 2D limit cycle in state space. Parameters for models: α1 = 1, β1 = 10−7, γ1 = 1.2, φ1 = 100, K = 106, r1 = 1. Initial conditions (A1-A2, B1-B2): Cx,0 = 105, G0 = 0, V0 = 0. Initial conditions (A3-A4, B3-B4): Cx,0 = 105, G0 = 105, V0 = 0. (TIF) [file pcbi.1010925.s005.tif]

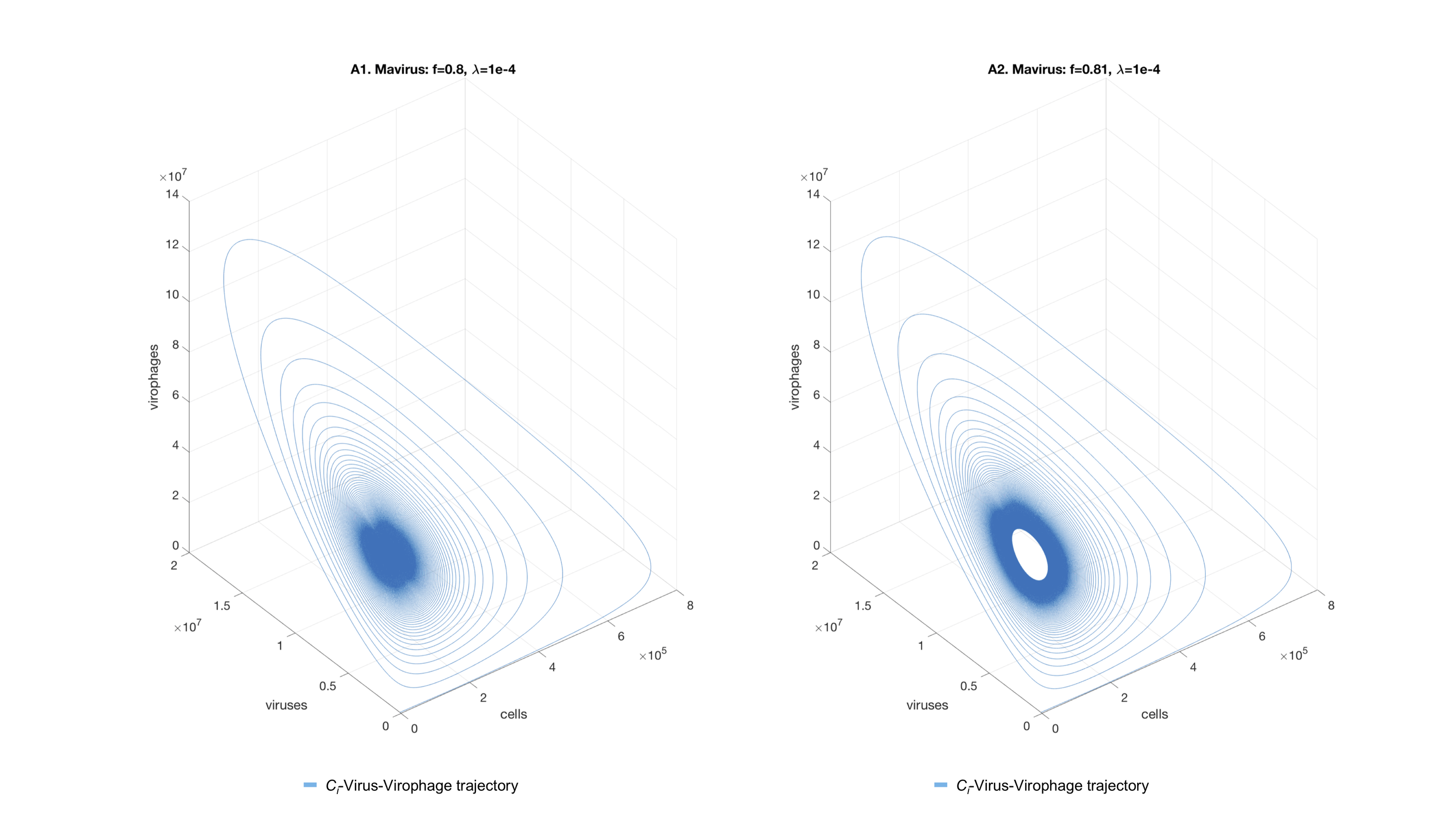

Supplement: S2 Fig — A1) When the inhibition parameter (f) is below the critical value of 0.8073, trajectories converge to a point equilibrium. A2) When f is increased over the critical value, trajectories converge to a limit cycle. (TIF) [file pcbi.1010925.s006.tif]

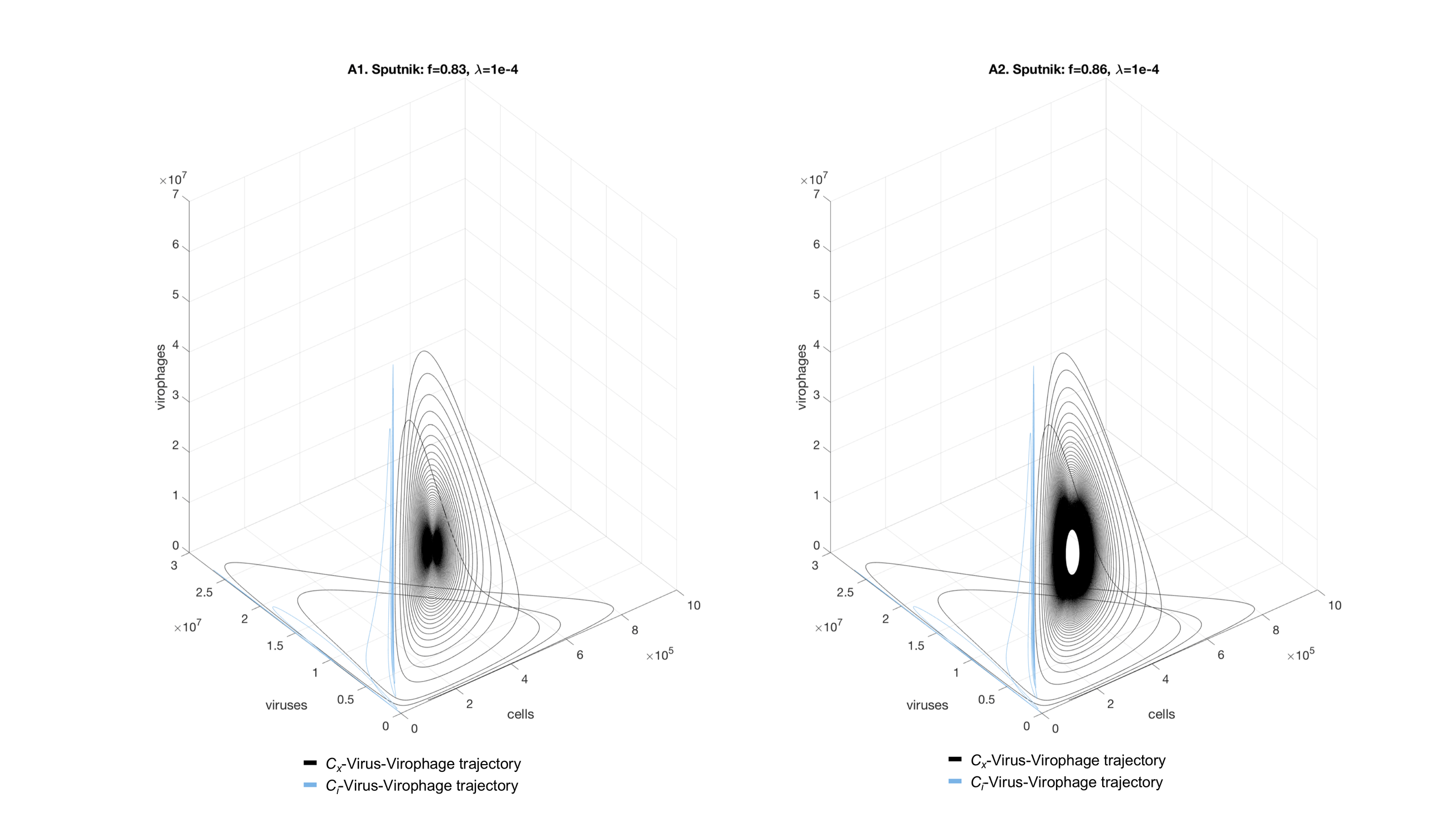

Supplement: S3 Fig — A1) When the inhibition parameter (f) is below the critical value of 0.8303, trajectories converge to a point equilibrium. A2) When f is increased over the critical value, trajectories converge to a limit cycle. (TIF) [file pcbi.1010925.s007.tif]

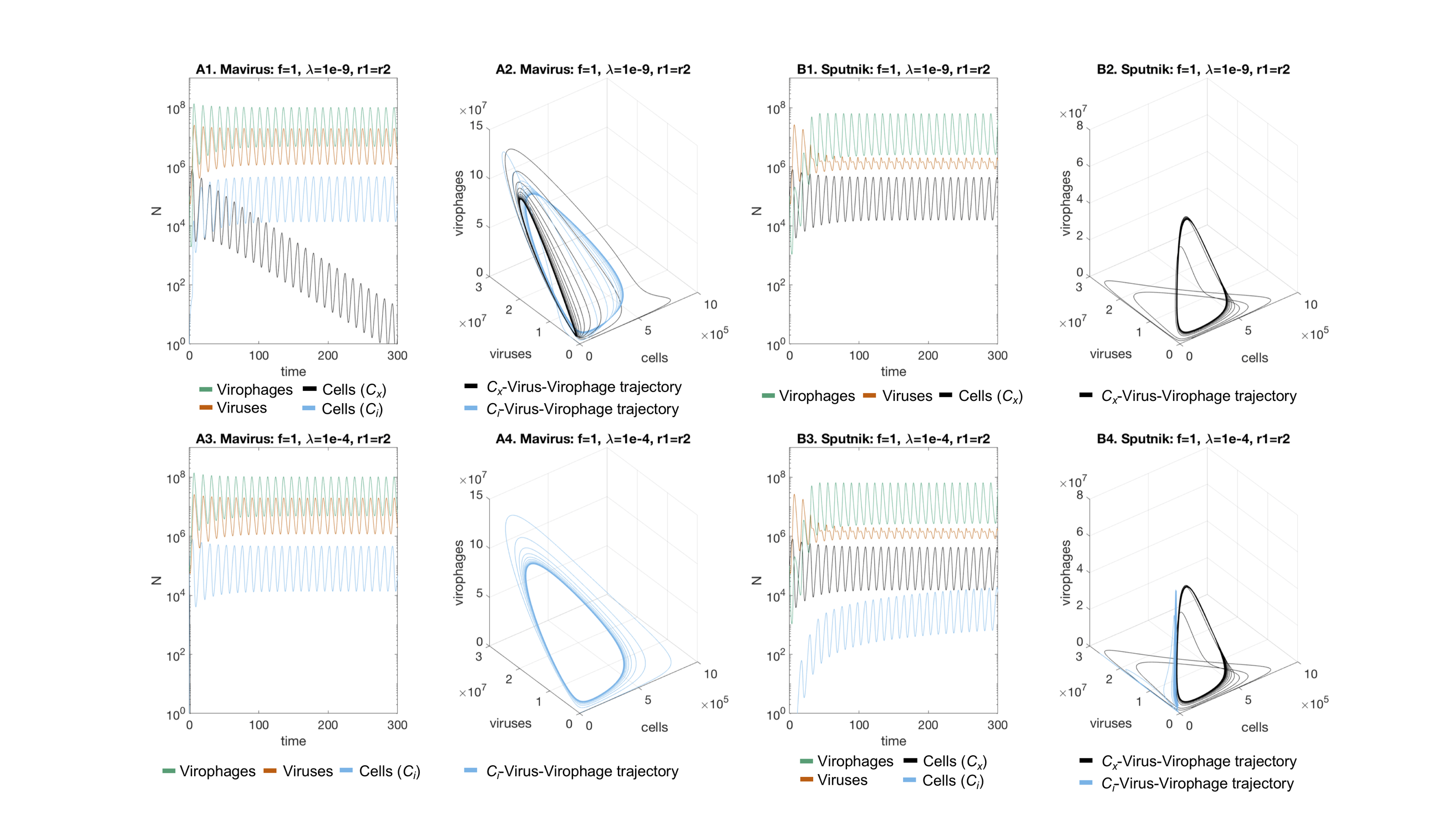

Supplement: S4 Fig — A. In the Mavirus model, cells with a provirophage become fixed at low (A1-A2) and high (A3-A4) integration rates. B. In the Sputnik model, naïve cells are observed for low integration (B1-B2), while coexistence is observed at a higher integration rate (B3-B4). Parameters for model 1: α1 = 1, α2 = 0.9, β1 = 10−7, β2 = 10−6, γ1 = 1.2, γ2 = 2.6, λ = {1e-9, 1e-4}, φ1 = 100, φ2 = 1000, f = 1, K = 106, r1 = 1, r2 = 1. Parameters for model 2 are the same except for β2 = 10−7 and k = 8⋅10−7. Initial conditions: Cx,0 = 105, G0 = 105, V0 = 105. (TIF) [file pcbi.1010925.s008.tif]

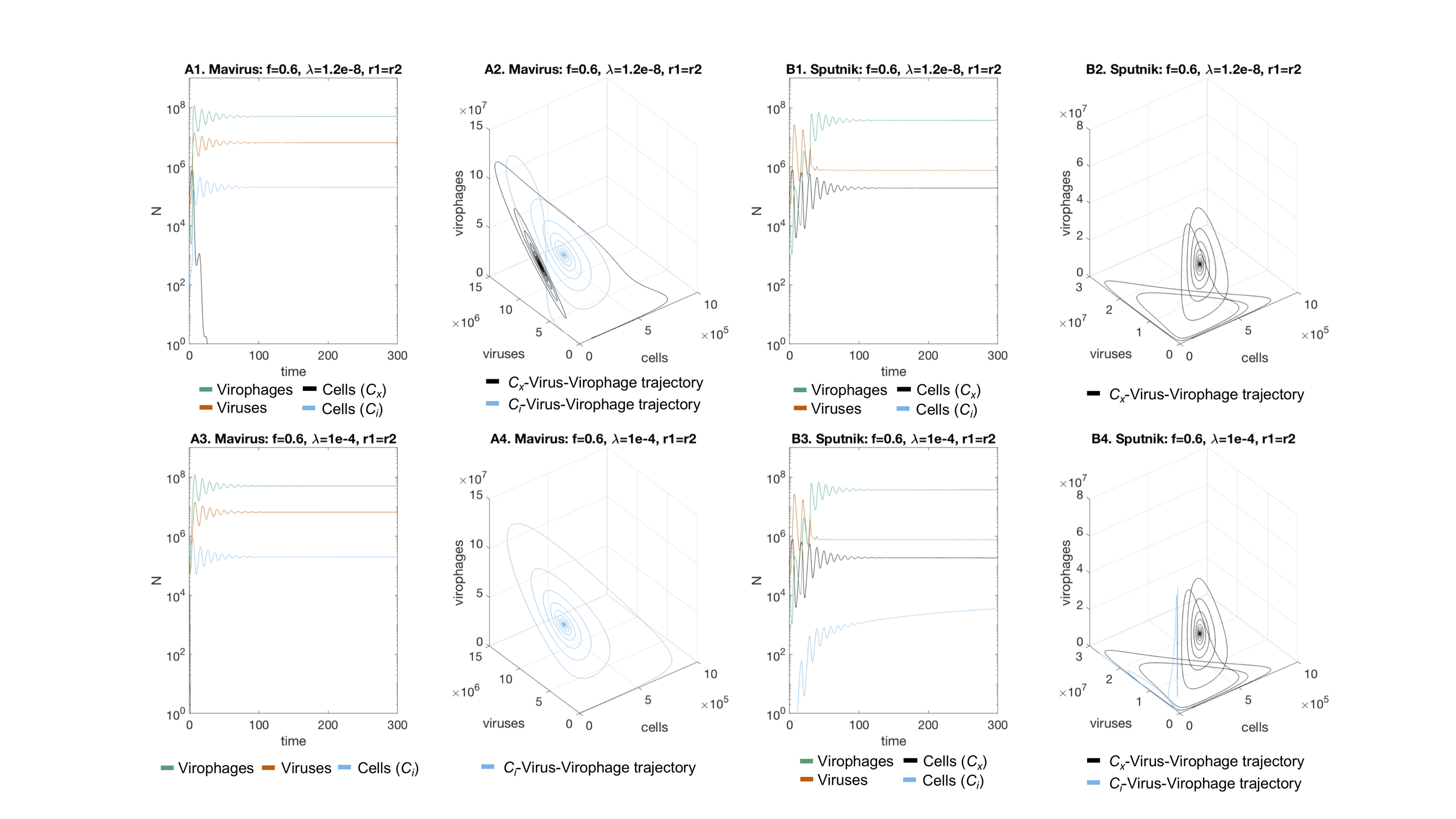

Supplement: S5 Fig — A. In the Mavirus model, cells with a provirophage become fixed at low (A1-A2) and high (A3-A4) integration rates. B. In the Sputnik model, naïve cells are observed for low integration (B1-B2), while coexistence is observed at a higher integration rate (B3-B4). Parameters for model 1: α1 = 1, α2 = 0.9, β1 = 10−7, β2 = 10−6, γ1 = 1.2, γ2 = 2.6, λ = {1.2e-8, 1e-4}, φ1 = 100, φ2 = 1000, f = 0, K = 106, r1 = 1, r2 = 1. Parameters for model 2 are the same except for β2 = 10−7 and k = 8⋅10−7. Initial conditions: Cx,0 = 105, G0 = 105, V0 = 105. (TIF) [file pcbi.1010925.s009.tif]

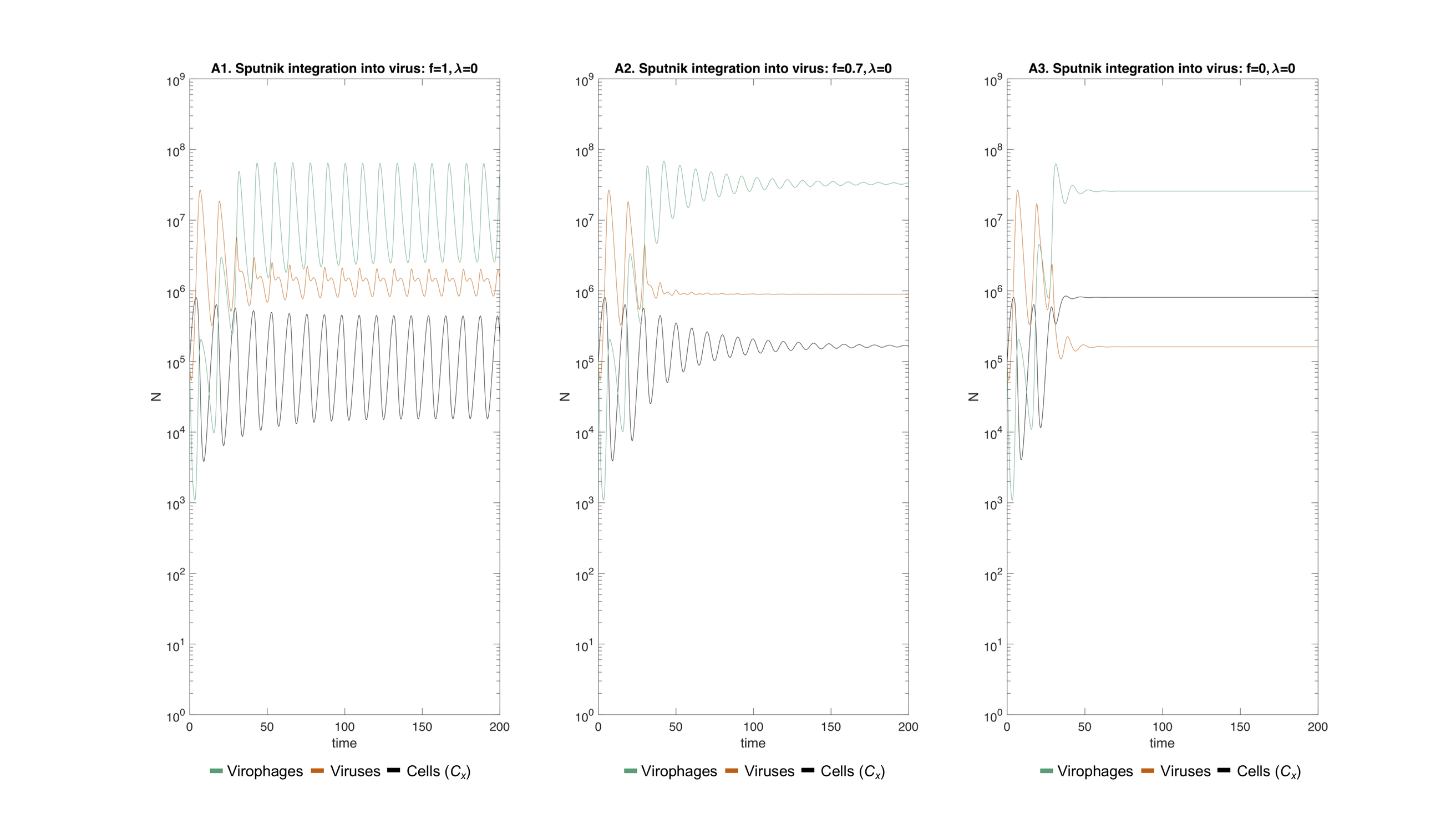

Supplement: S6 Fig — A1. Neutral virophages lead to a stable oscillatory regime. A2-A3. Virophage inhibition f = {0.7, 0} leads to loss of oscillations and stabilisation of the dynamics. Model parameters: α1 = 1, α2 = 0.9, β1 = 10−7, β2 = 10−7, γ1 = 1.2, γ2 = 2.6, φ1 = 100, φ2 = 1000, f = {1, 0.7, 0}, K = 106, r1 = 1, r2 = 0.8, λ* = 0. (TIF) [file pcbi.1010925.s010.tif]

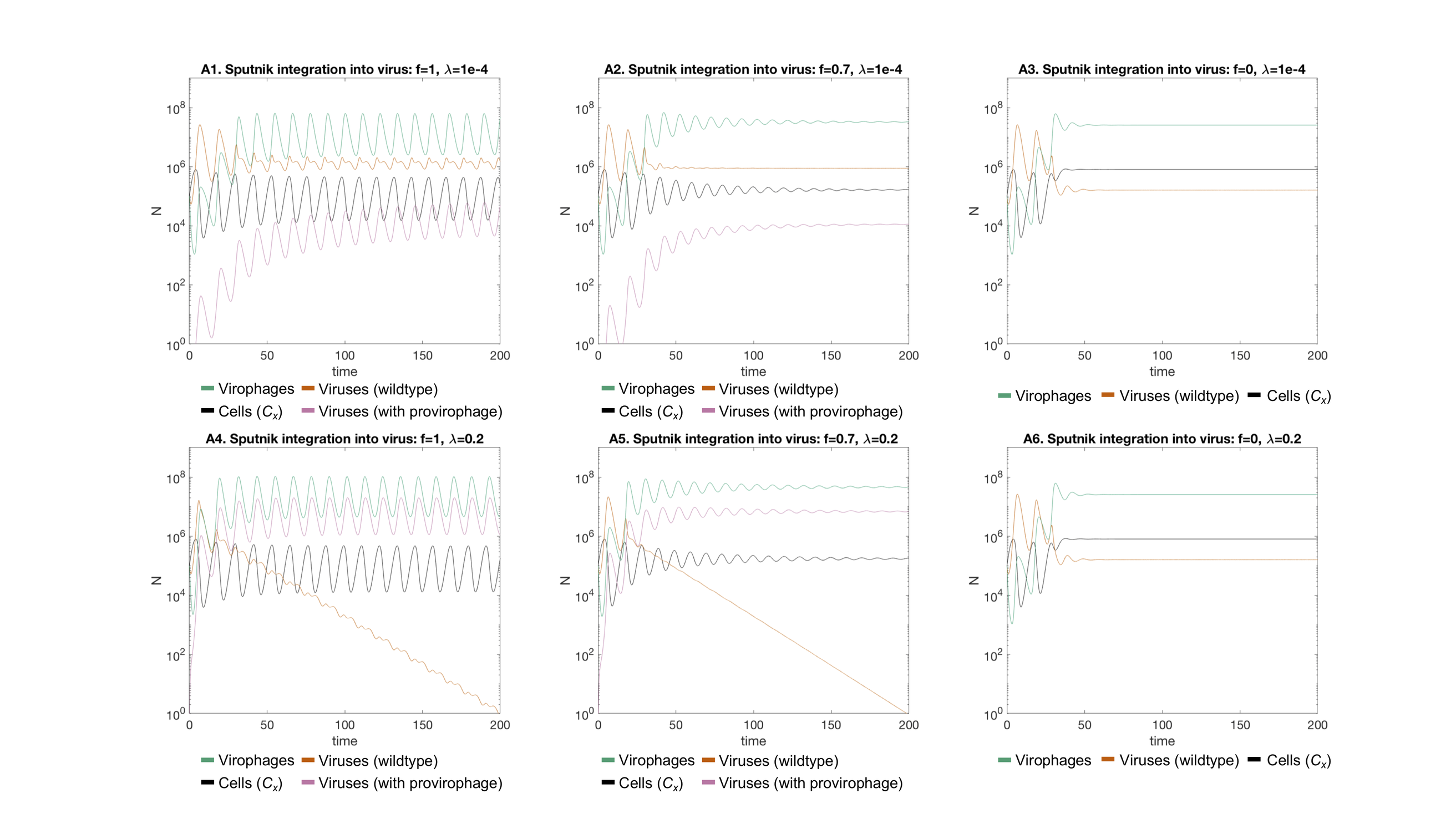

Supplement: S7 Fig — Virophage inhibition leads to stabilisation of the dynamics. A1-A3. In the presence of moderate integration (λ* = 10−4), viruses with an integrated virophage can establish in the population and are maintained as a polymorphism (f = {1, 0.7}), unless there is total virophage inhibition (f = 0). A4-A6. In the presence of high integration (λ* = 0.2), the original virus population is replaced by viruses carrying an integrated virophage (f = {1, 0.7}), except when inhibition is total (f = 0). Model parameters: α1 = 1, α2 = 0.9, β1 = 10−7, β2 = 10−7, γ1 = 1.2, γ2 = 2.6, φ1 = 100, φ2 = 1000, f = {1, 0.7, 0}, K = 106, r1 = 1, r2 = 0.8, λ = {10−4, 0.2}. (TIF) [file pcbi.1010925.s011.tif]

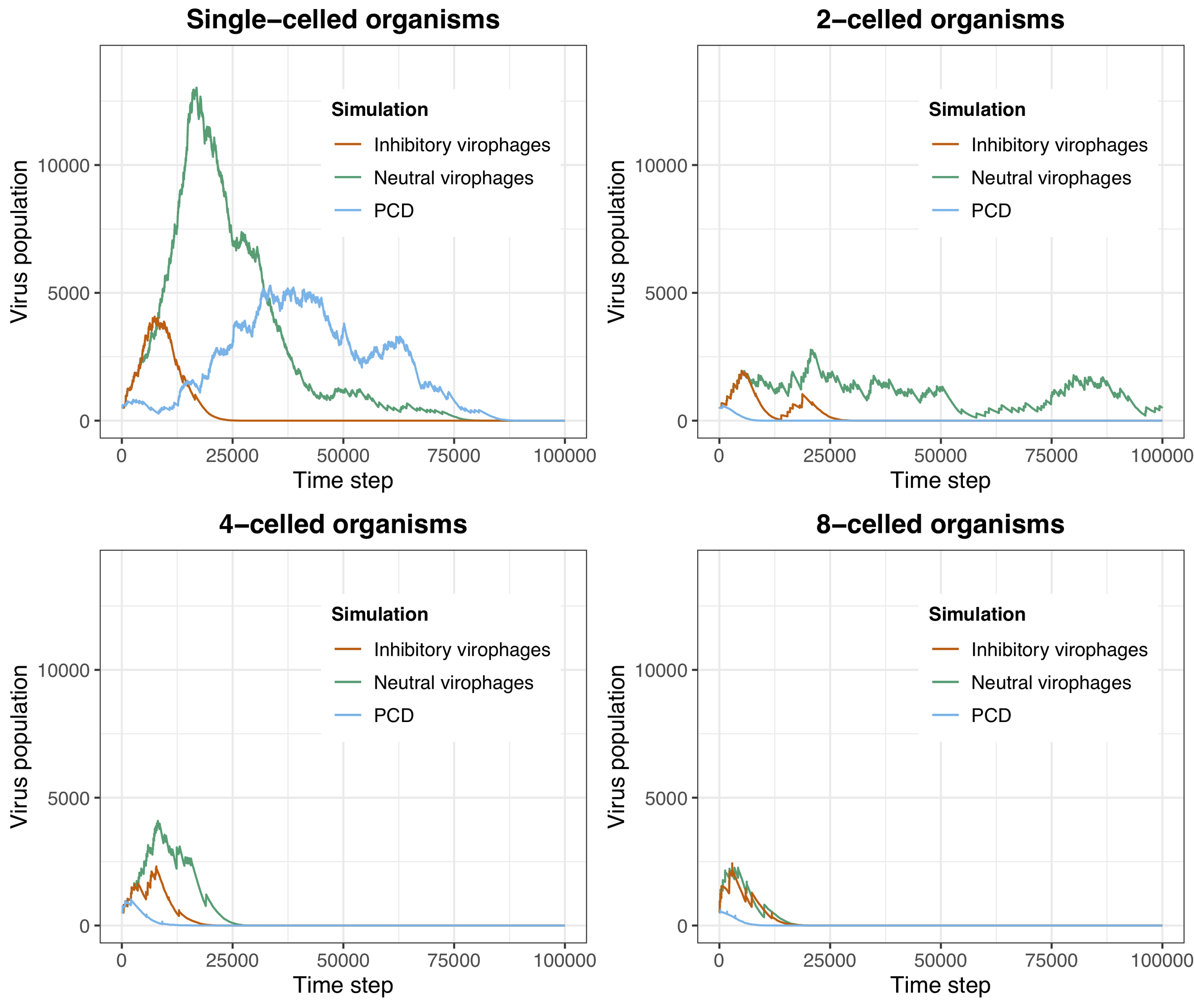

Supplement: S8 Fig — The total virus population during a stochastic simulation is shown as a function of time and grouped by the number of cells per organism and simulation. The highest virus wave is observed in the population of single-celled organisms in the presence of neutral virophages. By comparison, a simulation with inhibitory virophages and PCD show lower maxima of the virus waves. The effect of multicellularity can also be observed in the lower virus waves from the single-celled to 8-celled cases. Random seed = 1234. (TIF) [file pcbi.1010925.s012.tif]

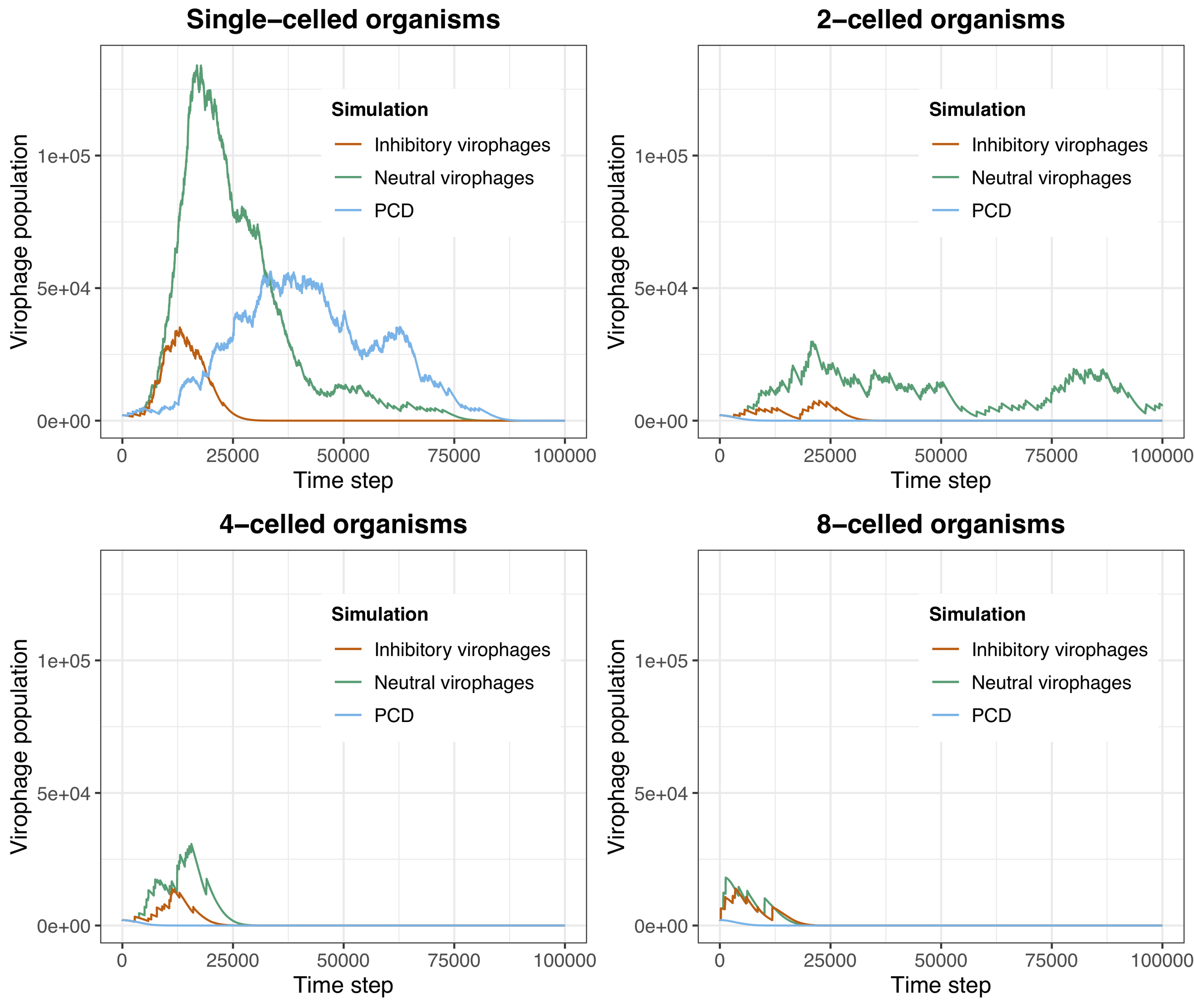

Supplement: S9 Fig — The total virophage population during a stochastic simulation is plotted as a function of time and grouped by the number of cells per organism and simulation. We observe the same pattern as for the virus population, where the highest virophage numbers were attained in the simulation with neutral virophages and lower for inhibitory virophages and PCD. In contrast to viruses, virophages attain much larger population sizes. Multicellularity also has an effect on reducing the maximum size of the virophage waves. Random seed = 1234. (TIF) [file pcbi.1010925.s013.tif]

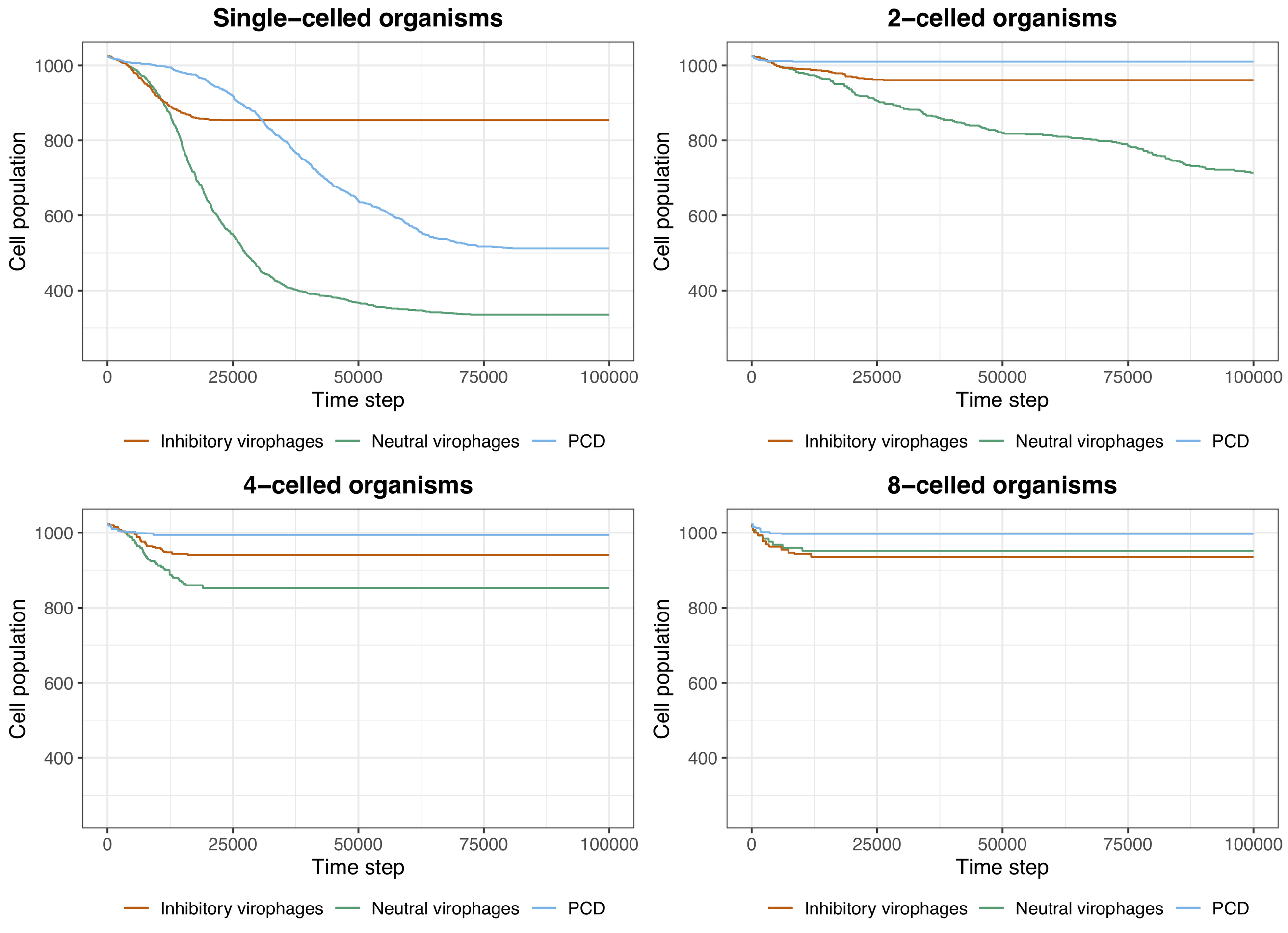

Supplement: S10 Fig — The total number of cells during a stochastic simulation is shown as a function of time and grouped by the number of cells per organism and simulation. We can observe a greater survival of cells in simulations with PCD and inhibitory virophages, while the lowest survival occurred in the simulations with neutral virophages. Grouping cells together in space also provided an advantage since the survival of cells increases with increasing number of cells per organism. Random seed = 1234. (TIF) [file pcbi.1010925.s014.tif]

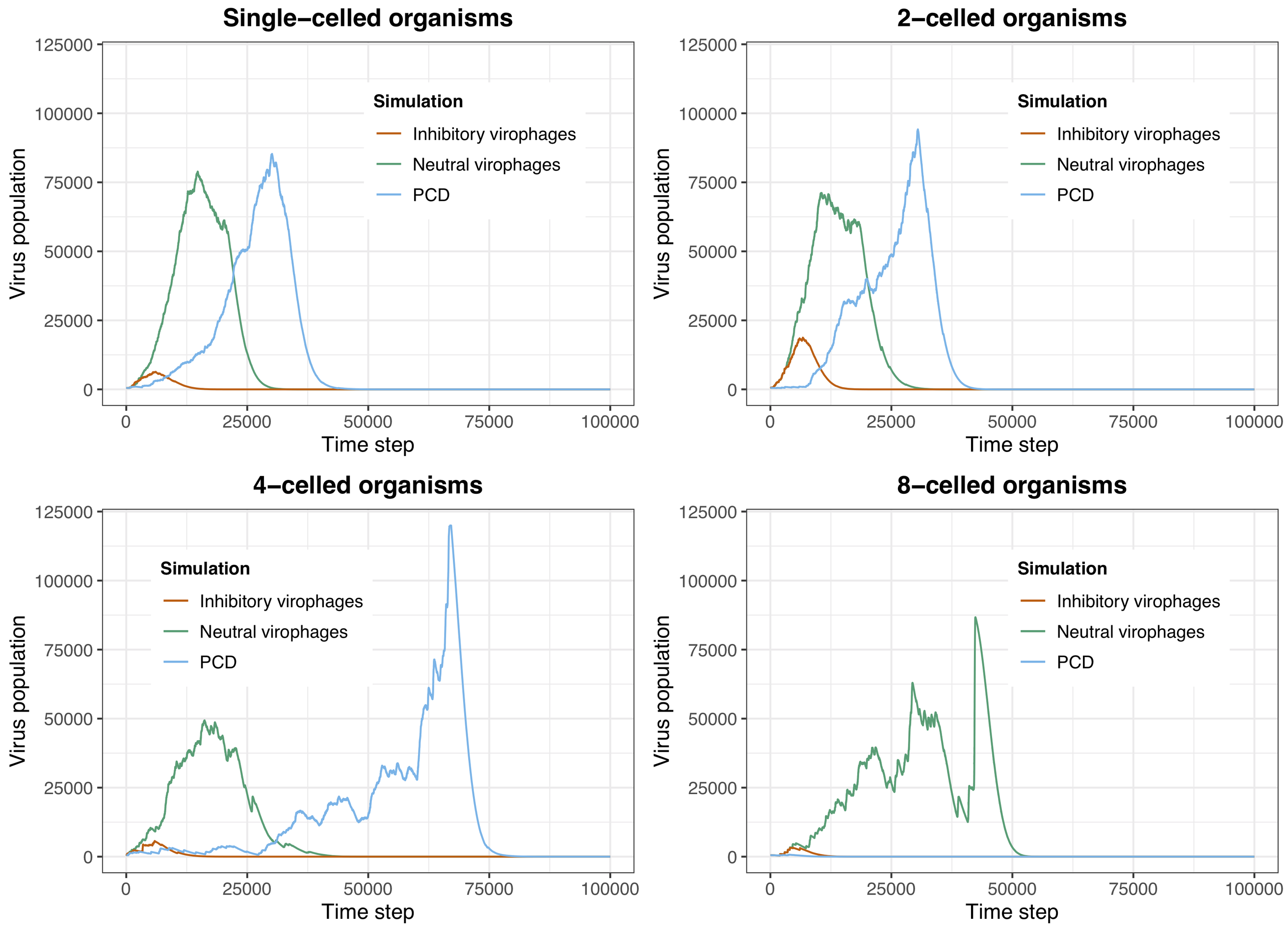

Supplement: S11 Fig — The total virus population during a stochastic simulation is shown as a function of time and grouped by the number of cells per organism and simulation. The highest virus wave is observed in the population of single-celled organisms in the presence of neutral virophages. The simulation with inhibitory virophages show lower maxima of the virus waves. The effect of multicellularity can also be observed in the lower virus waves from the single-celled to 8-celled cases. Random seed = 1234. (TIF) [file pcbi.1010925.s015.tif]

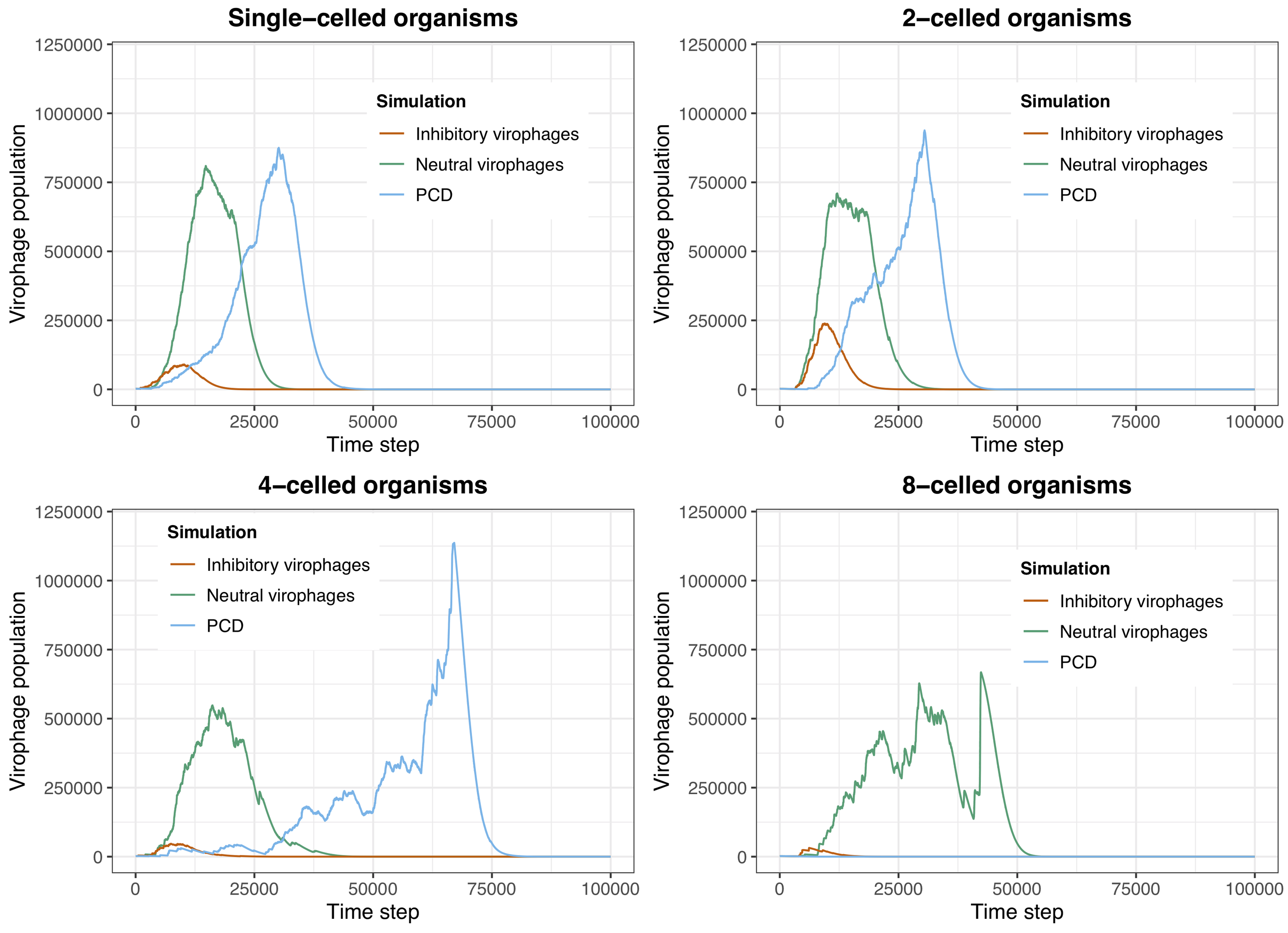

Supplement: S12 Fig — The total virophage population during a stochastic simulation is plotted as a function of time and grouped by the number of cells per organism and simulation. We observe the same pattern as for the virus population, where the highest virophage numbers were attained in the simulation with neutral virophages and lower for inhibitory virophages. In contrast to viruses, virophages attain much larger population sizes. Multicellularity also has an effect on reducing the maximum size of the virophage waves. Random seed = 1234. (TIF) [file pcbi.1010925.s016.tif]

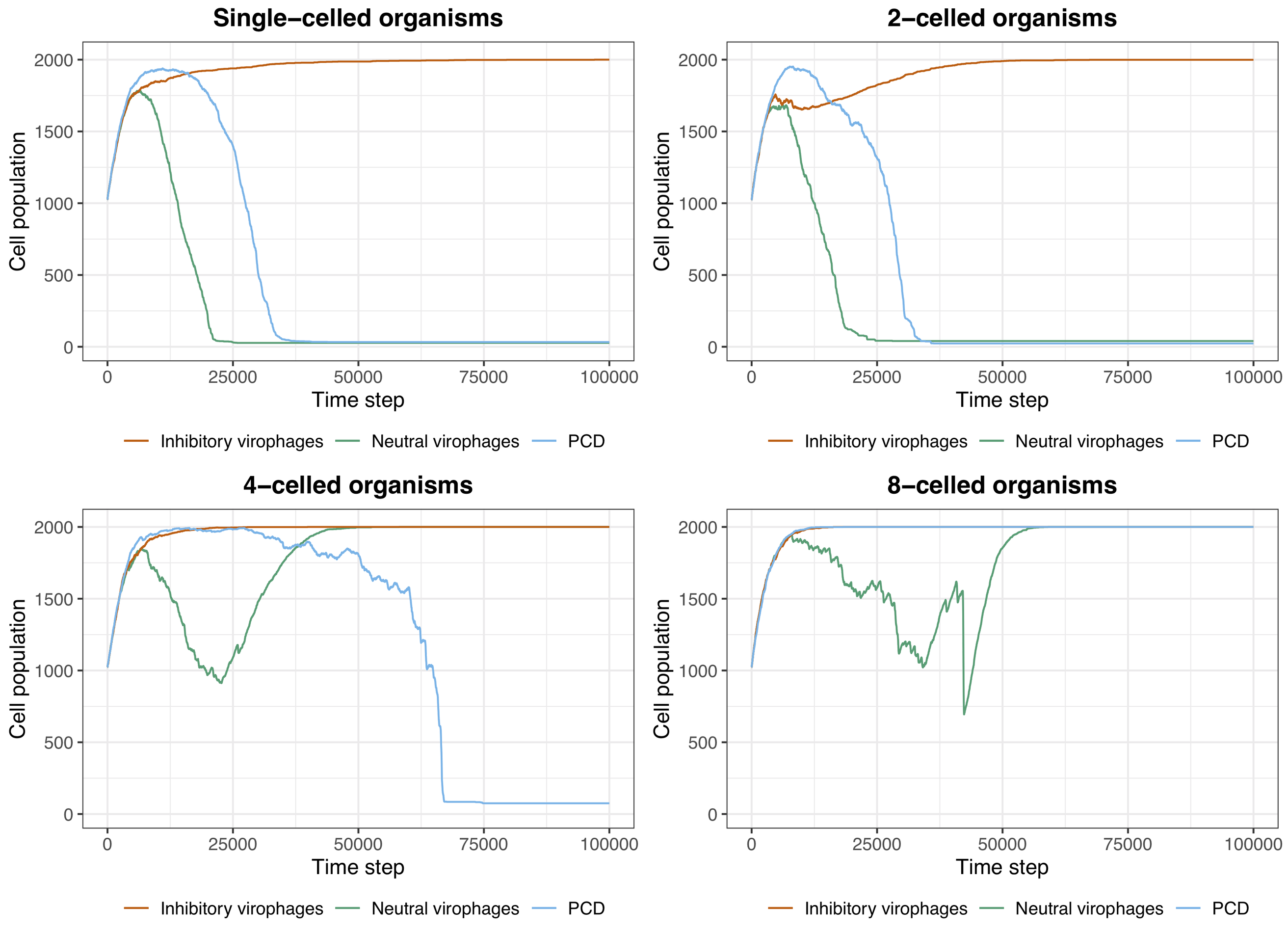

Supplement: S13 Fig — The total number of cells during a stochastic simulation is shown as a function of time and grouped by the number of cells per organism and simulation. We can observe a greater survival of cells in simulations with inhibitory virophages. Grouping cells together also provided an advantage since the survival of cells increases with increasing number of cells per organism across simulations. Random seed = 1234. (TIF) [file pcbi.1010925.s017.tif]
